# Comprehensive Analysis to Identify Key Genes Involved in Advanced Atherosclerosis

**DOI:** 10.1155/2021/4026604

**Published:** 2021-12-10

**Authors:** Tian-ming Huo, Zhi-wei Wang

**Affiliations:** Department of Cardiovascular Surgery, Renmin Hospital of Wuhan University, Wuhan, 430060 Hubei Province, China

## Abstract

**Background:**

The study was aimed at finding accurate and effective therapeutic targets and deepening our understanding of the mechanisms of advanced atherosclerosis (AA).

**Methods:**

We downloaded the gene expression datasets GSE28829, GSE120521, and GSE43292 from Gene Expression Omnibus. Weighted gene coexpression network analysis (WGCNA) was performed for GSE28829, and functional enrichment analysis and protein–protein interaction network analysis were conducted on the key module. Significant genes in the key module were analyzed by molecular complex detection, and genes in the most important subnetwork were defined as hub genes. Multiple dataset analyses for hub genes were conducted. Genes that overlapped between hub genes and differentially expressed genes (DEGs) of GSE28829 and GSE120521 were defined as key genes. Further validation for key genes was performed using GSE28829 and GSE43292. Gene set enrichment analysis (GSEA) was applied to key genes.

**Results:**

A total of 77 significant genes in the key module of GSE28829 were screened out that were mainly associated with inflammation and immunity. The subnetwork was obtained from significant genes, and 18 genes in this module were defined as hub genes, which were related to immunity and expressed in multiple diseases, particularly systemic lupus erythematosus. Some hub genes were regulated by SPI1 and associated with the blood, spleen, and lung. After overlapping with DEGs of GSE28829 and GSE120521, a total of 10 genes (HCK, ITGAM, CTSS, TYROBP, LAPTM5, FCER1G, ITGB2, NCF2, AIF1, and CD86) were identified as key genes. All key genes were validated and evaluated successfully and were related to immune response pathways.

**Conclusion:**

Our study suggests that the key genes related to immune and inflammatory responses are involved in the development of AA. This may deepen our understanding of the mechanisms of and provide valuable therapeutic targets for AA.

## 1. Background

Cardiovascular disease (CVD) is one of the leading causes of death in the world, and approximately 17 million people die from it every year [1]. Atherosclerosis (AS) is the most frequent cause of CVD [2]. The main feature of AS is complex chronic inflammation. Its pathogenesis and molecular mechanisms are multifactorial and characterized by smooth muscle cell proliferation, endothelial damage, cell apoptosis, inflammatory cell activation, lipid accumulation, vascular matrix changes, and oxidative stress [3, 4]. As early atherosclerosis (EA) progresses to advanced atherosclerosis (AA), atherosclerotic plaques will gradually expand and rupture, leading to vascular stenosis or occlusion and causing myocardial infarction and ischemic stroke [5, 6]. Current treatment strategies for reversing advanced plaque formation are still limited, and the mechanisms of AA are not fully elucidated [7]. Thus, more comprehensive and in-depth investigations of AA are needed.

In recent years, high-throughput platforms for gene expression analysis, such as microarrays, have become effective tools for revealing the pathogenesis of CVD. With the help of this method, researchers have already discovered many biomarkers related to CVD and AS. By comparing the expression levels of lncRNA from peripheral blood mononuclear cells in patients with coronary artery disease and healthy people, researchers found that ENST00000444488.1 and uc010yfd.1 can better distinguish these two groups [8]. NEDD4L, FBXO44, FBXO27, WSB1, FBXW8, UBE2F, and ASB1 have been reported as hub genes that help elucidate the pathogenesis and progression of AS [9]. Such studies have mainly been limited to plaque-related expression profiles, and only a few have addressed advanced plaque. Some scholars have found that the stability of atherosclerotic plaques is mainly affected by inflammation, matrix remodeling, and calcification, which are reflected in differentially expressed genes (DEGs) between stable plaque and unstable plaque [10]. By screening DEGs from stable and ruptured plaques, FABP4 and leptin have been shown to be involved in the process of atherosclerotic plaque rupture [11]. CCL4, CCL18, MMP9, and SPP1 are highly expressed in ruptured plaques and have been validated with experimental evidence [12].

However, some false positives may occur in these studies due to the limitation of the number of samples. While the hub genes are statistically significant, they are not functionally annotated in many cases, or they have important roles in the protein–protein interaction (PPI) network that is not statistically significant [13]. Also, because of the small rate of change or low abundance of some hub genes, information on those genes may have been missed.

Weighted gene coexpression network analysis (WGCNA) is a method that is mainly based on the network. Using this method, a scale-free network is constructed by analyzing all the expression profiles included in a study. WGCNA can identify gene coexpression network modules, determine the correlation between modules and phenotypes, and then discover important genes that regulate key biological processes [14]. This method has helped researchers achieve many remarkable results in numerous areas, including cancers [15], the nervous system [16], and the immune system [17].

In our study, we identified key modules and significant genes using WGCNA for GSE28829 and conducted a bioinformatic analysis for key module and hub genes to reveal potential functions. In order to avoid both false positive and false negative results, the DEGs of GSE28829 and GSE120521 were screened out to confirm some key genes. Finally, those key genes were validated in GSE28829 and GSE43292. We believe that this research can deepen our understanding of the mechanisms of AA and guide us in finding better treatment strategies.

## 2. Materials and Methods

### 2.1. Study Design

**Figure 1:**
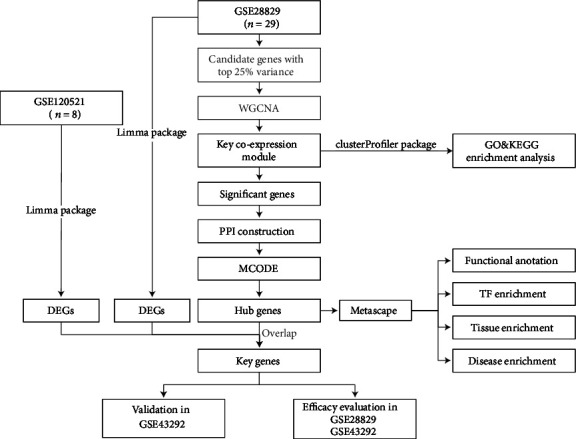
Research design flow chart.

The raw data for GSE28829 and GSE43292 and the normalized data for GSE120521 were downloaded from the Gene Expression Omnibus (GEO) database. The overall research design flow chart is shown in Figure 1. First, gene expression profiles of plaques in GSE28829 and GSE120521 were used to identify DEGs in EA and AA. For WGCNA, the variances of every gene in all samples were calculated and sorted in descending order, and the top 25% of genes were selected as candidates. Then, we selected a significant coexpression module for further analysis. To reveal the potential functions of the genes in this module, we used the clusterProfiler package for functional enrichment analysis. Moreover, significant genes were screened from this module based on gene significance and module membership (MM) value. Next, we constructed the PPI network and performed molecular complex detection (MCODE) analysis using the Cytoscape software to obtain the important subnetworks of these genes. The hub genes from the most important subnetwork were further analyzed with the Metascape tool. In order to find the key genes, we simultaneously mapped the hub genes to DEGs of GSE28829 and GSE120521, and the overlapping genes were identified as key genes. All of the key genes were validated in dataset GSE43292 and evaluated in datasets GSE28829 and GSE43292. Finally, to obtain further insights into the functions of these key genes, we performed a gene set enrichment analysis (GSEA) for each key gene.

### 2.2. Data Preprocessing

The three datasets were downloaded from the GEO database. The data consisted of gene expression profiles with early and advanced samples of carotid atheroma plaque. Details are shown in Table 1.

All analyses using R packages were based on the R software (version 4.0.2). The raw data of GSE28829 [18] and GSE43292 [19] were read using the affy package [20]. In order to make the data better for analysis, the robust multiarray average (RMA) method was used to normalize the data, and batch effects were removed [21]. When the probe expression data were duplicated, their average value was used as the gene expression value. For GSE120521 [10], the processed gene expression data provided by GEO were used.

### 2.3. Identification of DEGs

The limma R package was used to screen DEGs [22]. To calculate the *P* values of the genes, an adjusted *t*-test was used. The false discovery rate (FDR) was calculated using the Benjamini and Hochberg method. Genes with an FDR lower than 0.05 and an absolute value of fold change (FC) higher than 2 were set as significant DEGs.

### 2.4. Construction of Coexpression Network

In order to avoid the background noise owning to the low expression levels of genes, variances of every gene in samples of both groups in GSE28829 were calculated. The results were sorted in descending order, and the genes with the top 25% variance were selected as candidate genes. The coexpression network of the candidate genes was constructed with the WGCNA package [23], with the minimum number of genes in a module set at 50. The maximum number of genes in a module was set as the number of all input genes. The cut height was set at 0.2 to merge possible similar modules.

### 2.5. Identification of Key Modules Related to AA

The module eigengene (ME) represents the gene expression levels in the module. The relationship between the module and AA can be determined by calculating the correlation between the ME and the clinical phenotype of AS, and the module with the highest absolute value of ME is the key module. Gene significance (GS) represents the correlation between a gene and a phenotype. In the module trait correlation analysis, genes with an MM higher than 0.9 and a GS higher than 0.4 in the key module were identified as significant genes.

### 2.6. Functional Enrichment Analysis of the Key Module

In order to further understand the functions of the genes in the key module, the clusterProfiler package [24] was used to identify and visualize the gene ontology (GO) terms enriched by the genes in the key module. Significant enrichment was screened as a *P* value lower than 0.05.

### 2.7. PPI Network Construction of Significant Genes and Hub Gene Selection

The STRING database 11.5 was used to construct the PPI network of significant genes [25], and the combined score was chosen as greater than 0.4. The PPI network was visualized using the Cytoscape software (version 3.8.2) [26]. We screened the subnetworks of the PPI network using the MCODE plug-in [27]. The criteria used for cutoff were degree cutoff = 2, node score cutoff = 0.2, max depth = 100, and *K* − core = 2. The genes in the most significant subnetwork were defined as hub genes.

### 2.8. Multidatabase Analysis of Hub Genes

Metascape is a comprehensive portal containing 40 databases that integrate functions such as functional enrichment analysis, gene annotation, and interactive group analysis. Following the screening of the important subnetwork, the Metascape tool was used for further gene annotation analysis. Also, another three datasets, DisGeNET, PaGenBase, and TRRUST, were applied to identify the gene related disease, specific tissue, and transcription factor, respectively. The criteria for cutoff were set as *P* value < 0.01, enrichment factor > 1.5, and minimum count = 3.

### 2.9. Key Gene Selection and Validation

In order to avoid false positive rates in the results, hub genes were simultaneously mapped to DEGs of GSE28829 and GSE120521, and the overlapping genes were identified as key genes. All of the key genes were further validated in GSE28829 and GSE43292 databases. The pROC package [28] was used to plot the ROC curve and calculate the area under curve (AUC). The ROC curve was used to evaluate whether the key genes can distinguish AA and EA plaques well.

### 2.10. GSEA for Key Genes

GSEA was performed based on the gene list obtained from each key gene using the GSEA function from the R package clusterProfiler [29]. The reference gene set was h.all.v7.4.entrez.gmt in the Molecular Signatures Database. The criterion for significance was set as an adjusted *P* value < 0.05.

## 3. Results

### 3.1. Data Preprocessing and Identification of DEGs

**Figure 2:**
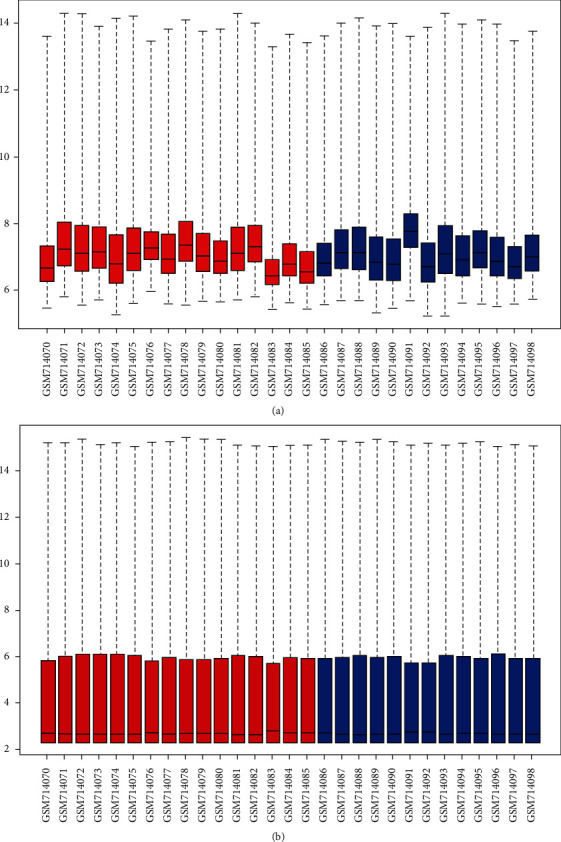
Box plots for the gene expression data. Red bars represent advanced atherosclerotic plaque samples, and blue bars represent early atherosclerotic plaque samples. The black lines in each box represent the median gene expression level. (a) The black lines of raw data are not at the same level. (b) After data processing, the black lines are almost at the same level.

The gene expression distribution of samples in GSE28829 before data processing is shown in Figure 2(a). We could see that their median distribution was not on a straight line. After normalization, the median value of gene expression was basically at the same level (Figure 2(b)). After that, a total of 329 DEGs were distinguished from GSE28829. Among these, 270 upregulated genes and 59 downregulated genes were screened out. The expression of genes in GSE120521 was already normalized. Next, 539 upregulated genes and 557 downregulated genes were screened out from GSE120521. The DEGs of the two datasets are shown in Table S1 and Table S2.

### 3.2. Coexpression Network Selection and Identification of the Significant Module

**Figure 3:**
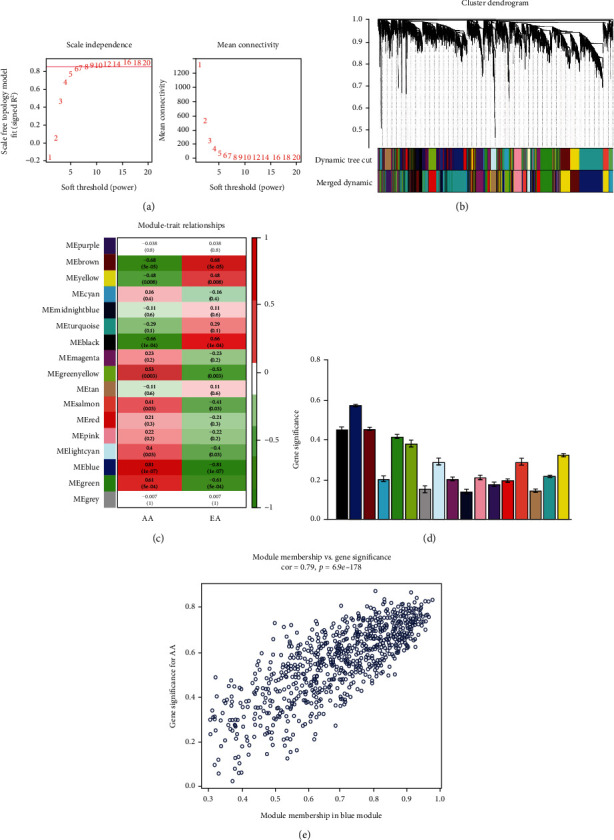
Gene coexpression networks in samples of GSE28829 and module trait correlation analysis of key coexpression network. (a) Analysis of the scale-free index for soft-thresholding powers and 0.85 were used as the correlation coefficient threshold, and the best soft-thresholding power was 7. (b) Gene dendrogram and modules colors of 29 samples in GSE28829. (c) Heatmap of the correlation between modules and AA and EA. (d) Module significance values of all the 17 coexpression modules associated with AA. (e) The gene significance for AA in the blue module.

A total of 5,044 genes out of 20,174 annotated genes were selected as candidates with the top 25% variance. In order to gain further insight into the biological functions of these genes in the progression of AA, we conducted the WGCNA analysis. The network was built using the WGCNA R package. After calculation, the best soft-thresholding power was set at 7, and the correlation coefficient threshold was set at 0.85 (Figure 3(a)). Several modules comprised most genes, which can be seen with the blue, brown, green, and yellow-green areas in Figure 3(b). The relationship between a module and a phenotype was analyzed, and multiple modules were related to AA (Figure 3(c)). The GS of all genes in each module is shown in Figure 3(d). We could intuitively see that the blue one is the module that has the most significant relationship with AA.  Figure 3(e) provides the relationship between the MM and GS of each gene in the blue module. A total of 77 genes with a high GS for AA were selected as the hub genes.

### 3.3. Functional Enrichment Analysis of the Key Coexpression Module

**Figure 4:**
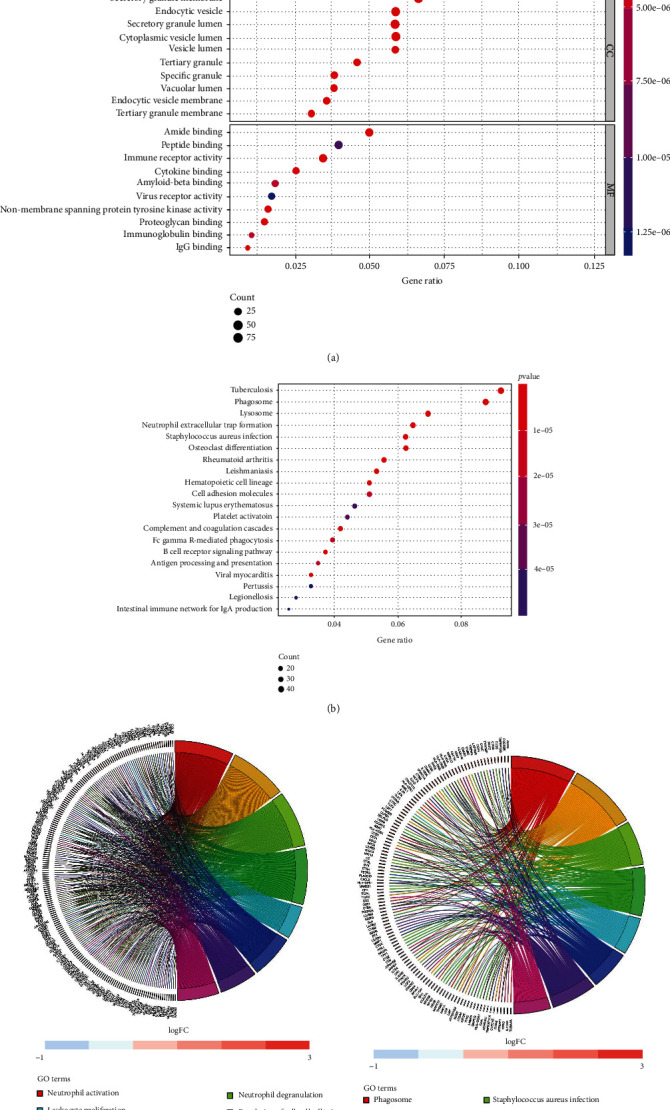
Functional enrichment analysis for blue module of AA plaque. (a) Biological process, cellular component, and molecular function analysis. (b) KEGG pathway analysis. (c) The relationship between genes and GO terms. (d) The relationship between genes and KEGG terms.

The GO terms of the biological process (BP) analysis showed that the BPs of the blue module were mainly enriched in neutrophil activation, neutrophil activation involved in immune response, neutrophil-mediated immunity, neutrophil degranulation, and leukocyte migration, which are indicative of immune cell stimulation and migration in patients with AA (Figure 4(a)). The GO terms of the cellular component (CC) were mainly enriched in the secretory granule membrane, endocytic vesicle, secretory granule lumen, and vesicle lumen. The GO terms of molecular function (MF) were enriched in amide binding, peptide binding, immune receptor activity, cytokine binding, and amyloid beta binding. The Kyoto Encyclopedia of Genes and Genomes (KEGG) pathway is mainly involved in tuberculosis, phagosome, lysosome, neutrophil extracellular trap formation, osteoclast differentiation, rheumatoid arthritis, leishmaniasis, hematopoietic cell lineage, cell adhesion molecules, and systemic lupus erythematosus. In addition, these AA correlated pathways were related to immunity–inflammation responses (Figure 4(b)).

The relationship between those genes and BP terms indicated that many genes enriched in neutrophil activation are also related to other BP terms, such as immune response, leukocyte proliferation, leukocyte migration, and regulation of cell–cell adhesion, which indicates that those genes could be related to multiple biological pathways involved in the progression of AA (Figure 4(c)). Also, Figure 4(d) shows that many genes related to phagosome, tuberculosis, and *Staphylococcus aureus* infection are also enriched for other pathways, including leishmaniasis, rheumatoid arthritis, and osteoclast differentiation.

Overall, these findings demonstrate that genes in the blue module are involved in immune and inflammation-related functions.

### 3.4. PPI Construction and Multidatabase Analysis of Modules

**Figure 5:**
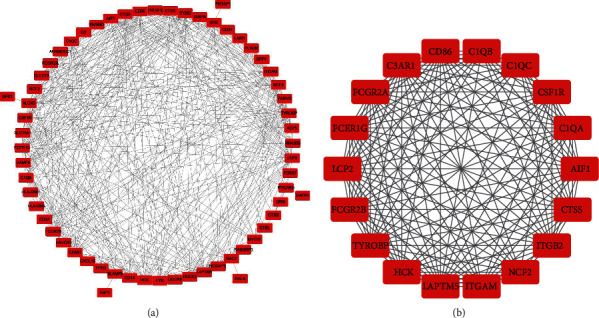
The PPI network of blue module and significant subnetwork of network. (a) Protein-protein interaction (PPI) network of the significant genes in blue module after WGCNA. (b) Subnetwork of the module with highest scores in PPI network after MCODE.

The PPI network of the significant genes in the blue module (interaction score > 0.4) was constructed, and 61 nodes and 398 interaction pairs were identified from the network (Figure 5(a)). Two highly connected modules were harvested by the MCODE analysis, and only one module had a score greater than 10 (16.941) (Figure 5(b)). The module contained 18 nodes and 144 edges. The genes in the module 1 were identified as hub genes.

**Figure 6:**
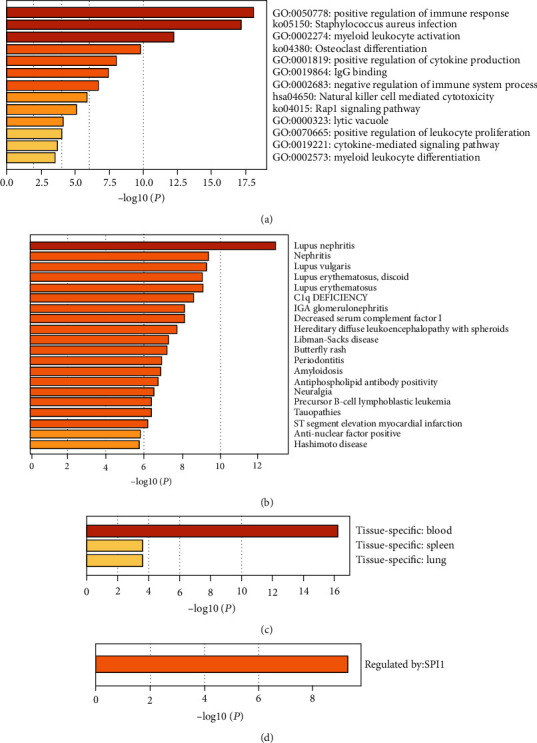
Multidatabase analysis of hub genes. (a) Biological functions of hub genes. (b) Disease enrichment related to hub genes involved in AA. (c) Enrichment of genes in specific tissues. (d) Enrichment of transcriptional regulators of hub genes.

In order to fully understand the role hub genes play in the development of AA, we conducted a multidatabase analysis of these genes. Enrichment analysis of module 1 in the Metascape database indicated that the hub genes are mainly related to positive regulation of immune response, *Staphylococcus aureus* infection, myeloid leukocyte activation, osteoclast differentiation, positive regulation of cytokine production, IgG binding, negative regulation of the immune system process, natural killer cell mediated cytotoxicity, Rap1 signaling pathway, lytic vacuole, positive regulation of leukocyte proliferation, cytokine-mediated signaling pathway, and myeloid leukocyte differentiation (Figure 6(a)). Disease enrichment analysis in the DisGeNET database revealed that these genes were mainly related to lupus nephritis, nephritis, lupus vulgaris, and lupus erythematosus (Figure 6(b)). Tissue characteristic enrichment analysis in the PaGenBase database suggested that hub genes were enriched in the blood, spleen, and lung (Figure 6(c)). Transcription factors analysis in the TRRUST database showed that the hub genes were mainly regulated by SPI1 (Figure 6(d)).

### 3.5. Key Gene Selection and Validation

**Figure 7:**
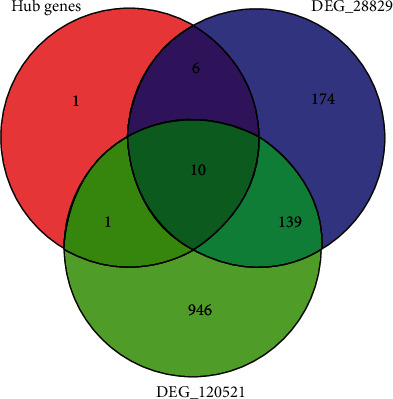
Key genes selection. A total of 10 common genes related to hub genes and DEGs of GSE28829 and GSE120521.

In order to find the key genes, the hub genes were mapped to the DEGs from GSE28829 and GSE120521 (Figure 7), and 10 genes were screened out: HCK, ITGAM, CTSS, TYROBP, LAPTM5, FCER1G, ITGB2, NCF2, AIF1, and CD86.

**Figure 8:**
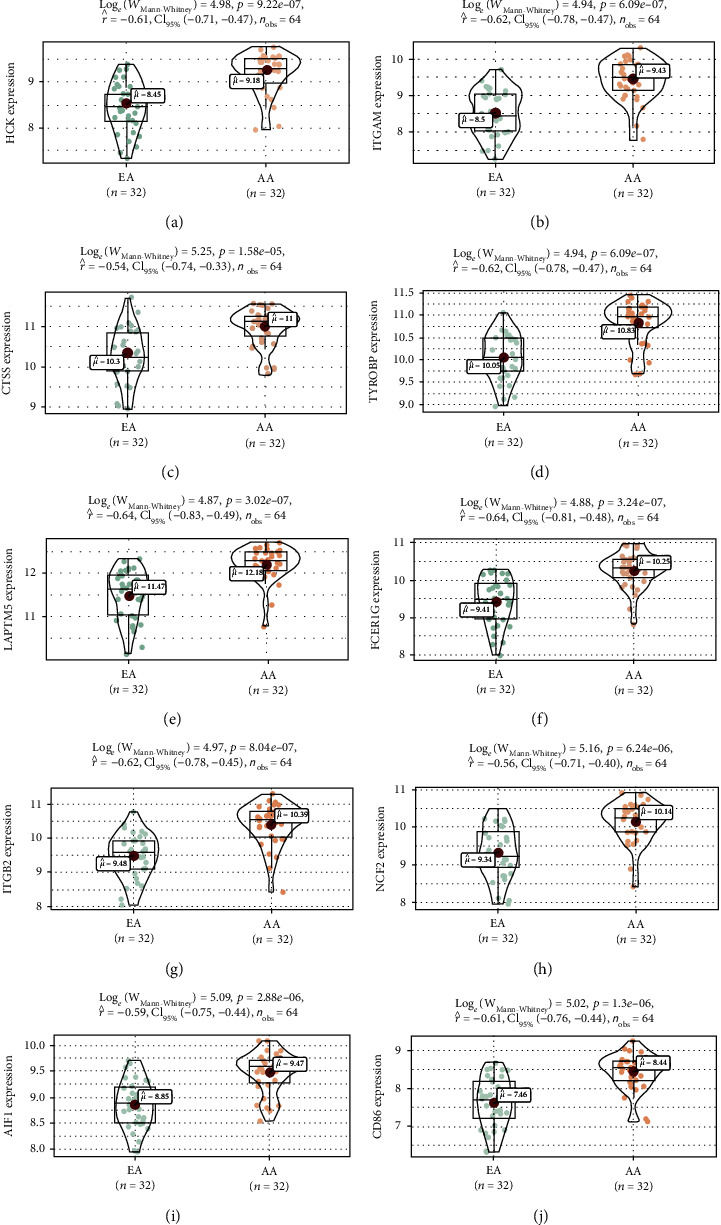
Validation of key genes in dataset GSE43292. (a) HCK. (b) ITGAM. (c) CTSS. (d) TYROBP. (e) LAPTM5. (f) FCER1G. (g) ITGB2. (h) NCF2. (i) AIF1. (j) CD86.

**Figure 9:**
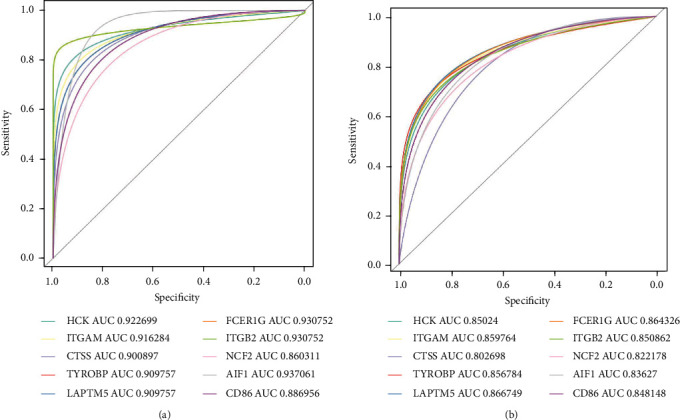
ROC curve of key genes. (a) ROC curve for GSE28829. (b) ROC curve for GSE43292.

The expression levels of all 10 of the key genes were tested in GSE43292. The results (Figure 8) showed that all of the key genes were highly expressed (all *P* < 0.001) in AA plaques as compared to EA in GSE43292. Furthermore, we plotted the ROC and calculated the AUCs for the key genes and found that all of the AUCs of key genes were greater than 0.8 in GSE28829 (Figure 9(a)) and GSE43292 (Figure 9(b)).

### 3.6. Gene Set Enrichment Analysis

**Figure 10:**
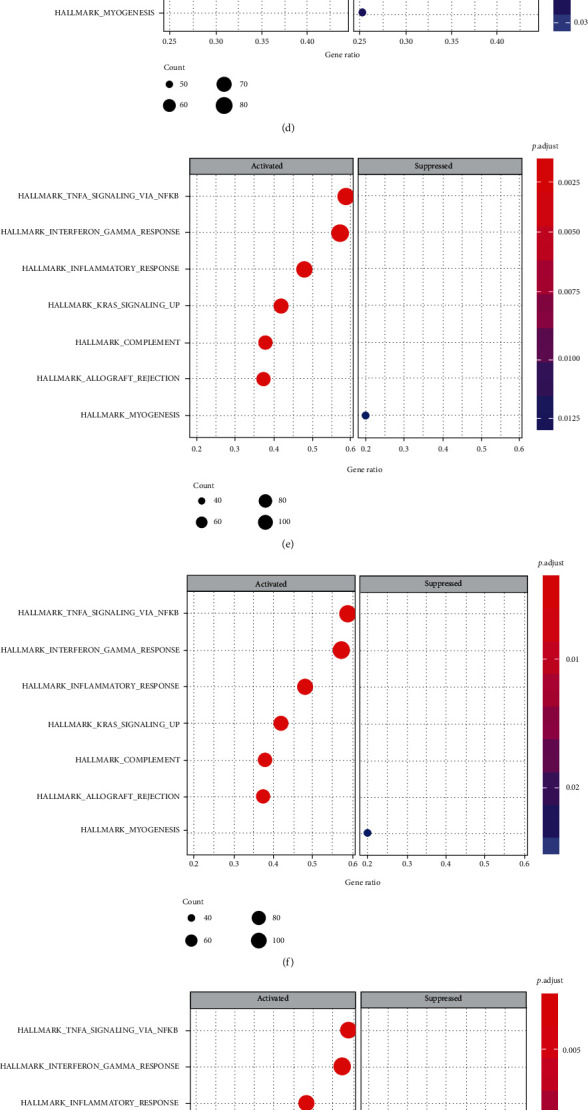
Gene set enrichment analysis for key genes. (a) HCK. (b) ITGAM. (c) CTSS. (d) TYROBP. (e) LAPTM5. (f) FCER1G. (g) ITGB2. (h) NCF2. (i) AIF1. (j) CD86.

**Figure 11:**
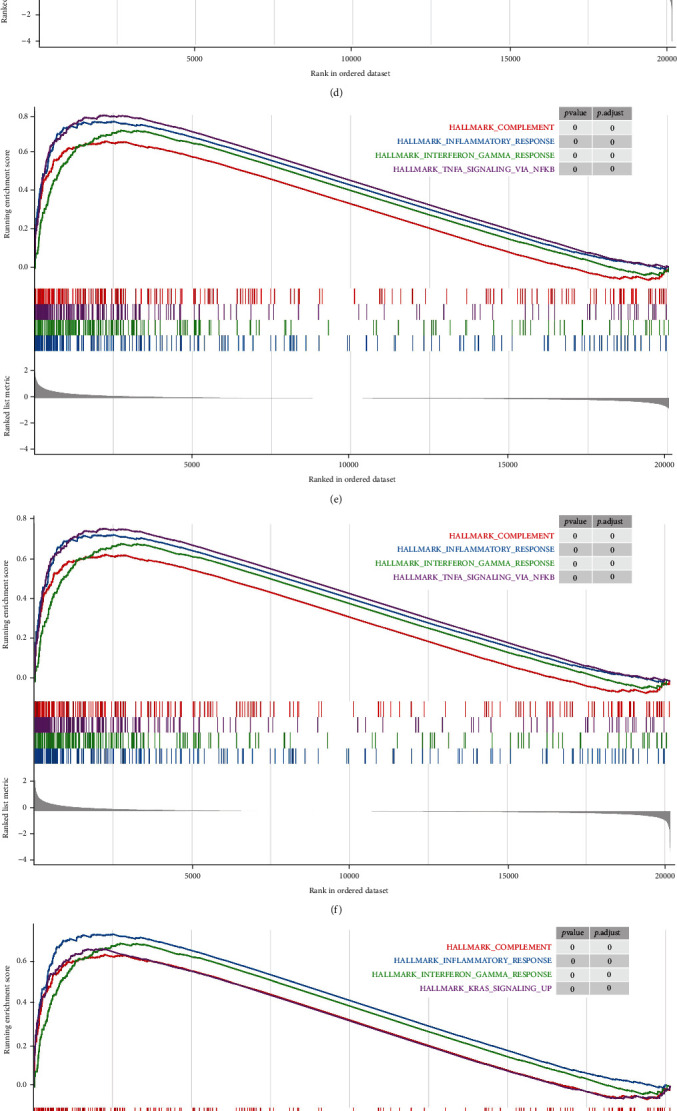
Gene sets related to immunity for key genes. (a) HCK. (b) ITGAM. (c) CTSS. (d) TYROBP. (e) LAPTM5. (f) FCER1G. (g) ITGB2. (h) NCF2. (i) AIF1. (j) CD86.

The full list of gene sets enriched in AA plaques with those 10 key genes was highly expressed using GSEA (Figure 10). All of the gene sets were mainly related to immunity and inflammation. In addition, AIF1 (Figure 10(i)) was also related to oxidative phosphorylation and allograft rejection. The gene sets related to immunity and inflammation were selected to perform further analysis. The gene sets “complement,” “inflammatory response,” “interferon *γ* response,” and “TNF-*α* signaling via NF-*κ*B” were enriched in samples with high expression levels of HCK, ITGAM, CTSS, TYROBP, LAPTM5, FCER1G, ITGB2, NCF2, and CD86 (Figure 11). The samples with high AIF1 expression were mainly enriched in “complement,” “inflammatory response,” and “interferon *γ* response” (Figure 11(i)).

## 4. Discussion

Our study applied WGCNA to build the gene network related to AA and found some coexpression networks. Combined with PPI and MCODE plug-in analyses, several key genes associated with the pathogenesis of AA were identified. Our findings broaden the horizons of the mechanism of AS development from early to advanced stages.

In the present study, through the WGCNA analysis, 17 coexpression modules were determined. The blue module, containing 829 genes, was most significantly associated with AA. We performed an enrichment analysis on the genes in the blue module and found that the blue module was mainly enriched in immune and inflammatory pathways. In recent decades, a lot of research has been conducted to examine the immune and inflammatory mechanisms in AS. Researchers have found that inflammation is closely related to AS and plaque instability [30]. Monocyte-differentiated macrophages become foam cells after ingesting lipids, and foam cells can cause cell adhesion, matrix degradation, and inflammatory cell infiltration by secreting inflammatory factors, which can lead to plaque rupture [31]. The activation of neutrophils can be affected by oxidatively modified low-density lipoprotein (oxLDL), thereby enhancing the formation of a neutrophil extracellular trap (NET). After the formation of NET, the enzymes released by neutrophils can induce the oxidative modification and/or degradation of LDL, produce modified proinflammatory LDL, and promote the further activation of neutrophils [32]. NET can also aggravate endothelial dysfunction, causing plaque instability and weakening of the fibrous cap, leading to AS and thrombosis [33]. Th1 cells mainly promote inflammation, while Th2 cells show a dual role not only slightly promoting the occurrence of AS but also inhibiting the development of AS [34]. Regulatory T cells mainly inhibit the formation of AS [35, 36]. Therefore, our research also confirmed that immune cells are involved in the formation of AA plaques.

In order to find genes that are more closely related to AA in the key module, we selected 77 significant genes from the blue module for further analysis by setting the MM and GS values. By constructing the PPI network, we harvested the subnetwork with the highest score from significant genes, which had a total of 18 hub genes. We conducted a Metascape analysis, and the results showed that the genes are related to many biological functions, including positive regulation of immune response, *Staphylococcus aureus* infection, myeloid leukocyte activation, and osteoclast differentiation. These biological functions are also related to immune cells, which further confirms the role of immune response in AA. According to the results of the TRRUST database analysis, SPI1 is the main regulatory transcription factor for these genes. It has been reported that the expression of SPI1 increased during the differentiation process of myeloid cells, while the expression in differentiated mast cells, monocytes, B cells, and peripheral blood neutrophils maintained high levels [37]. The DisGeNET database analysis showed that these genes are closely related to systemic lupus erythematosus (SLE) and other diseases. The common point of SLE and AS is inflammation as the main feature, and the difference is that the inflammation of SLE is autoimmune, which impairs several organ systems, including the cerebrovascular and cardiovascular systems [38, 39]. The enhanced proinflammatory state and systemic inflammation play an important role during the formation of atherosclerotic thrombosis [38]. AS may also be accelerated by systemic inflammation. Therefore, the prevalence of AS in SLE patients is greater than that in the general population [40]. Tissue characteristic enrichment analysis indicated that these genes were enriched in the blood, spleen, and lung. It also revealed that the tissue distribution of genes has a strong correlation with immunity. The multidatabase analysis further confirmed that the hub gene related immune and inflammatory response plays an important role in AA and helped us to understand the pathogenesis of AA from more aspects.

We also incorporated the DEGs of GSE28829 and GSE120521 for combined analysis. Finally, we found 10 key genes: HCK, ITGAM, CTSS, TYROBP, LAPTM5, FCER1G, ITGB2, NCF2, AIF1, and CD86. ITGAM and ITGB2 encode the *α*M chain and *β*2 chain of integrin, respectively. Under the stimulation of inflammation and thrombus, the *α*M *β*2 integrin can mediate the adhesion of neutrophils and monocytes to endothelial cells [41]. HCK is a signal transduction protein that mainly transmits membrane receptor signals. It plays an important role in the innate immune response by regulating the phagocytosis of neutrophils and the proliferation and migration of macrophages [42]. It is reported that after knocking out HCK, the endothelial adhesion and migration in AS plaques will be weakened, leading to decreases in plaque formation [43]. Interestingly, the same study found that after HCK knockout, monocytes had a subpopulation imbalance and accumulated under the endothelium, which increases the instability of the plaque. Our research showed that the high levels of HCK expression in AA are a risk factor for the further development of AS. We estimate that the different effects may be related to different stages of AS, such as the difference between AA and EA. Whether it protects or aggravates AS and what its mechanism needs to be further explored. TYROBP, also known as DAP12, is a transmembrane receptor widely found in neutrophils and monocytes/macrophages [44, 45]. Studies have found that DAP12 seems to be related to lipid deposition and plaque inflammation in the process of promoting AS [46]. CTSS can degrade antigen proteins into peptides and can also reshape the components of the extracellular matrix [47]. Previous studies have shown that CTSS is expressed by endometrial macrophages and smooth muscle cells and that it participates in the formation of AS, together with serine proteases and MMP [48]. NCF2 is a neutrophil solute factor that encodes a subunit of NADPH oxidase [49]. NADPH oxidase is the main source of reactive oxygen species (ROS), which mainly play the roles of antibacterial, anti-inflammatory, and redox signal transduction [50]. Researchers pointed out that NADPH oxidase and the ROS produced are significantly related to hypertension [51]. Oxidative stress is a risk factor for AS. Some studies have suggested that NADPH may be involved in the promotion of atherosclerotic inflammation [52]. However, there is no direct evidence that NCF2 is involved in the progression of AS. AIF1 is mainly expressed in cells of the monocyte lineage [53]. In vitro, AIF1 can enhance the phagocytosis and lipid uptake of macrophages [54] and can also increase the proliferation and migration of macrophages, inducing inflammation [55]. LAPTM5 positively regulates proinflammatory signaling pathways by promoting NF-*κ*B and MAPK signaling and the production of proinflammatory cytokines in macrophages [56]. At present, FCER1G and CD86 are rarely studied in the cardiovascular system.

Our research showed that not only these key genes are significantly increased in AA plaques of GSE28829 but also, more importantly, their expression levels were verified in another dataset (GSE43292). We also found that these genes are mainly enriched in “complement,” “inflammatory response,” “interferon *γ* response,” and “TNF-*α* signaling via NF-*κ*B.” In fact, the roles of the above pathways in AS have been frequently studied. For example, TNF-*α* can guide inflammatory cells to accumulate in atherosclerotic plaques, affect plaque stability, and cause thrombosis and cell necrosis [57, 58]. And the plaque stability affection of TNF-*α* underlines its role in promoting the formation of AA. IFN*γ* affects immune cells, endothelial cells, and smooth muscle cells in AS plaques [59, 60]. This also further confirms the connection between these genes and immune cells, which is consistent with existing research.

As a whole, some previous studies have resolved the relationship between key genes and AS/AA, while our research further emphasized the relationships of genes and immune responses with AA, but the mechanism behind their involvement is still unclear.

Although we tried to find key genes through multiple algorithms and increase the credibility of these genes by using multiple datasets, our research still has certain limitations. First, the key genes are based on studies of AS and normal groups in most existing studies. Therefore, if the comparison to the normal group can be added to our study, we may be able to dynamically understand the roles of these genes in the whole process of AS, from initiation to development. In fact, we have tried to include samples of atherosclerosis at different stages in the study, but we did not find any suitable datasets. Also, we have not further studied the exact molecular mechanisms of key genes involved in AA. Furthermore, as mentioned above, immune response has become the main pathogenic factor of AA. The changes of immune cells at different stages of the disease and the relationship between immune cells and genes will become our research focus in the future.

## 5. Conclusion

In conclusion, we comprehensively discussed the cells and related factors involved in the development of AA. This further confirmed that, in AA, immune response has become the main pathogenic mechanism. It was also discovered that multiple key genes play an important role in the development of EA to AA. This deepens our understanding of the occurrence and development of AA and also provides a strong basis for us to find a treatment for the disease.

## Figures and Tables

**Table 1:** The summary of the datasets.

GEO ID	Platform	Tissue type	Sample size	Experiment type
GSE28829	GPL570	Advanced carotid atherosclerotic plaques and early carotid atherosclerotic plaques	16 vs. 13	Array
GSE120521	GPL16791	Unstable carotid atherosclerotic plaques and stable carotid atherosclerotic plaques	4 vs. 4	High-throughput sequencing
GSE43292	GPL6244	Advanced carotid atherosclerotic plaques and early carotid atherosclerotic plaques	32 vs. 32	Array

## Data Availability

The data used to support the findings of this study are available from the corresponding author upon request.

## Supplementary Materials

Supplementary Materials Table S1: the DEGs of GSE28829. Table S2: the DEGs of GSE120521.

## References

[1] Benjamin E. J., Muntner P., Alonso A. (2019). Heart Disease and Stroke Statistics-2019 update: a report from the American Heart Association. *Circulation*.

[2] Lee K. K., Stelzle D., Bing R. (2019). Global burden of atherosclerotic cardiovascular disease in people with hepatitis C virus infection: a systematic review, meta-analysis, and modelling study. *The Lancet Gastroenterology & Hepatology*.

[3] Weber C., Noels H. (2011). Atherosclerosis: current pathogenesis and therapeutic options. *Nature Medicine*.

[4] Singh R. B., Mengi S. A., Xu Y. J., Arneja A. S., Dhalla N. S. (2002). Pathogenesis of atherosclerosis: a multifactorial process. *Experimental and Clinical Cardiology*.

[5] Sakakura K., Nakano M., Otsuka F., Ladich E., Kolodgie F. D., Virmani R. (2013). Pathophysiology of atherosclerosis plaque progression. *Heart, Lung & Circulation*.

[6] Spagnoli L. G., Mauriello A., Sangiorgi G. (2004). Extracranial thrombotically active carotid plaque as a risk factor for ischemic stroke. *JAMA*.

[7] Gaurav C., Saurav B., Goutam R., Goyal A. K. (2015). Nano-systems for advanced therapeutics and diagnosis of atherosclerosis. *Current Pharmaceutical Design*.

[8] Li L., Wang L., Li H. (2018). Characterization of lncRNA expression profile and identification of novel lncRNA biomarkers to diagnose coronary artery disease. *Atherosclerosis*.

[9] Gu Y., Ma X., Li J., Ma Y., Zhang Y. (2021). Identification of candidate targets for the diagnosis and treatment of atherosclerosis by bioinformatics analysis. *American Journal of Translational Research*.

[10] Mahmoud A. D., Ballantyne M. D., Miscianinov V. (2019). The human-specific and smooth muscle cell-enriched lncRNA SMILR promotes proliferation by regulating mitotic CENPF mRNA and drives cell-cycle progression which can be targeted to limit vascular remodeling. *Circulation Research*.

[11] Lee K., Santibanez-Koref M., Polvikoski T., Birchall D., Mendelow A. D., Keavney B. (2013). Increased expression of fatty acid binding protein 4 and leptin in resident macrophages characterises atherosclerotic plaque rupture. *Atherosclerosis*.

[12] Chen P., Chen Y., Wu W., Chen L., Yang X., Zhang S. (2019). Identification and validation of four hub genes involved in the plaque deterioration of atherosclerosis. *Aging*.

[13] Shi W., Zou R., Yang M. (2019). Analysis of genes involved in ulcerative colitis activity and tumorigenesis through systematic mining of gene co-expression networks. *Frontiers in Physiology*.

[14] Zhang B., Horvath S. (2005). A general framework for weighted gene co-expression network analysis. *Statistical Applications in Genetics and Molecular Biology*.

[15] Wang Z., Ren Z., Li R. (2021). Multi-omics integrative bioinformatics analyses reveal long non-coding RNA modulates genomic integrity via competing endogenous RNA mechanism and serves as novel biomarkers for overall survival in lung adenocarcinoma. *Frontiers in Cell and Development Biology*.

[16] Mendez E. F., Wei H., Hu R. (2021). Angiogenic gene networks are dysregulated in opioid use disorder: evidence from multi-omics and imaging of postmortem human brain. *Molecular Psychiatry*.

[17] Yao M., Zhang C., Gao C. (2021). Exploration of the shared gene signatures and molecular mechanisms between systemic lupus erythematosus and pulmonary arterial hypertension: evidence from transcriptome data. *Frontiers in Immunology*.

[18] Döring Y., Manthey H. D., Drechsler M. (2012). Auto-antigenic protein-DNA complexes stimulate plasmacytoid dendritic cells to promote atherosclerosis. *Circulation*.

[19] Ayari H., Bricca G. (2013). Identification of two genes potentially associated in iron-heme homeostasis in human carotid plaque using microarray analysis. *Journal of Biosciences*.

[20] Gautier L., Cope L., Bolstad B. M., Irizarry R. A. (2004). affy--analysis of Affymetrix GeneChip data at the probe level. *Bioinformatics*.

[21] Irizarry R. A., Hobbs B., Collin F. (2003). Exploration, normalization, and summaries of high density oligonucleotide array probe level data. *Biostatistics*.

[22] Ritchie M. E., Phipson B., Wu D. (2015). limma powers differential expression analyses for RNA-sequencing and microarray studies. *Nucleic Acids Research*.

[23] Langfelder P., Horvath S. (2008). WGCNA: an R package for weighted correlation network analysis. *BMC Bioinformatics*.

[24] Yu G., Wang L. G., Han Y., He Q. Y. (2012). clusterProfiler: an R package for comparing biological themes among gene clusters. *OMICS*.

[25] Szklarczyk D., Gable A. L., Lyon D. (2019). STRING v11: protein-protein association networks with increased coverage, supporting functional discovery in genome-wide experimental datasets. *Nucleic Acids Research*.

[26] Shannon P., Markiel A., Ozier O. (2003). Cytoscape: a software environment for integrated models of biomolecular interaction networks. *Genome Research*.

[27] Bader G. D., Hogue C. W. (2003). An automated method for finding molecular complexes in large protein interaction networks. *BMC Bioinformatics*.

[28] Sing T., Sander O., Beerenwinkel N., Lengauer T. (2005). ROCR: visualizing classifier performance in R. *Bioinformatics*.

[29] Subramanian A., Tamayo P., Mootha V. K. (2005). Gene set enrichment analysis: a knowledge-based approach for interpreting genome-wide expression profiles. *Proceedings of the National Academy of Sciences of the United States of America*.

[30] Raggi P., Genest J., Giles J. T. (2018). Role of inflammation in the pathogenesis of atherosclerosis and therapeutic interventions. *Atherosclerosis*.

[31] Glass C. K., Witztum J. L. (2001). Atherosclerosis: The Road Ahead. *Cell*.

[32] Obama T., Itabe H. (2020). Neutrophils as a novel target of modified low-density lipoproteins and an accelerator of cardiovascular diseases. *International Journal of Molecular Sciences*.

[33] Soehnlein O. (2012). Multiple roles for neutrophils in atherosclerosis. *Circulation Research*.

[34] Daugherty A., Rateri D. L. (2002). T lymphocytes in atherosclerosis: the yin-yang of Th1 and Th2 influence on lesion formation. *Circulation Research*.

[35] Ley K. (2020). Role of the adaptive immune system in atherosclerosis. *Biochemical Society Transactions*.

[36] Saigusa R., Winkels H., Ley K. (2020). T cell subsets and functions in atherosclerosis. *Nature Reviews. Cardiology*.

[37] Wittwer J., Marti-Jaun J., Hersberger M. (2006). Functional polymorphism in ALOX15 results in increased allele-specific transcription in macrophages through binding of the transcription factor SPI1. *Human Mutation*.

[38] Hahn B. H. (2003). Systemic lupus erythematosus and accelerated atherosclerosis. *The New England Journal of Medicine*.

[39] Arkema E. V., Svenungsson E., Von Euler M., Sjowall C., Simard J. F. (2017). Stroke in systemic lupus erythematosus: a Swedish population-based cohort study. *Annals of the Rheumatic Diseases*.

[40] Roman M. J., Shanker B. A., Davis A. (2003). Prevalence and correlates of accelerated atherosclerosis in systemic lupus erythematosus. *The New England Journal of Medicine*.

[41] Mazzone A., Mazzucchelli I., Fossati G. (1996). Iloprost effects on phagocytes in patients suffering from ischaemic diseases: in vivo evidence for down-regulation of alpha M beta 2 integrin. *European Journal of Clinical Investigation*.

[42] Roseweir A. K., Powell A., Horstman S. L. (2019). Src family kinases, HCK and FGR, associate with local inflammation and tumour progression in colorectal cancer. *Cellular Signalling*.

[43] Medina I., Cougoule C., Drechsler M. (2015). Hck/Fgr kinase deficiency reduces plaque growth and stability by blunting monocyte recruitment and intraplaque motility. *Circulation*.

[44] Kobayashi M., Konishi H., Takai T., Kiyama H. (2015). A DAP12-dependent signal promotes pro-inflammatory polarization in microglia following nerve injury and exacerbates degeneration of injured neurons. *Glia*.

[45] Otero K., Turnbull I. R., Poliani P. L. (2009). Macrophage colony-stimulating factor induces the proliferation and survival of macrophages via a pathway involving DAP12 and *β*-catenin. *Nature Immunology*.

[46] Wang H. M., Gao J. H., Lu J. L. (2018). Pravastatin improves atherosclerosis in mice with hyperlipidemia by inhibiting TREM-1/DAP12. *European Review for Medical and Pharmacological Sciences*.

[47] Campden R. I., Zhang Y. (2019). The role of lysosomal cysteine cathepsins in NLRP3 inflammasome activation. *Archives of Biochemistry and Biophysics*.

[48] Sukhova G. K., Zhang Y., Pan J. H. (2003). Deficiency of cathepsin S reduces atherosclerosis in LDL receptor-deficient mice. *The Journal of Clinical Investigation*.

[49] Kenney R. T., Leto T. L. (1990). A HindIII polymorphism in the human NCF2 gene. *Nucleic Acids Research*.

[50] Breitenbach M., Rinnerthaler M., Weber M. (2018). The defense and signaling role of NADPH oxidases in eukaryotic cells: review. *Wiener Medizinische Wochenschrift (1946)*.

[51] Li H., Han X., Hu Z. (2018). Associations of NADPH oxidase-related genes with blood pressure changes and incident hypertension: the GenSalt study. *Journal of Human Hypertension*.

[52] Marques J., Cortes A., Pejenaute A., Zalba G. (2020). Implications of NADPH oxidase 5 in vascular diseases. *The International Journal of Biochemistry & Cell Biology*.

[53] Utans U., Quist W. C., McManus B. M. (1996). Allograft inflammatory factory-1. A cytokine-responsive macrophage molecule expressed in transplanted human hearts. *Transplantation*.

[54] Mishima T., Iwabuchi K., Fujii S. (2008). Allograft inflammatory factor-1 augments macrophage phagocytotic activity and accelerates the progression of atherosclerosis in ApoE-/- mice. *International Journal of Molecular Medicine*.

[55] Yang Z. F., Ho D. W., Lau C. K. (2005). Allograft inflammatory factor-1 (AIF-1) is crucial for the survival and pro-inflammatory activity of macrophages. *International Immunology*.

[56] Glowacka W. K., Alberts P., Ouchida R., Wang J. Y., Rotin D. (2012). LAPTM5 Protein Is a Positive Regulator of Proinflammatory Signaling Pathways in Macrophages. *The Journal of Biological Chemistry*.

[57] Koukkunen H., Penttilä K., Kemppainen A. (2001). C-reactive protein, fibrinogen, interleukin-6 and tumour necrosis factor-alpha in the prognostic classification of unstable angina pectoris. *Annals of Medicine*.

[58] Wang S. S., Hu S. W., Zhang Q. H., Xia A. X., Jiang Z. X., Chen X. M. (2015). Mesenchymal stem cells stabilize atherosclerotic vulnerable plaque by anti-inflammatory properties. *PLoS One*.

[59] McLaren J. E., Ramji D. P. (2009). Interferon gamma: a master regulator of atherosclerosis. *Cytokine & Growth Factor Reviews*.

[60] Tabas I., Lichtman A. H. (2017). Monocyte-macrophages and T cells in atherosclerosis. *Immunity*.

