# Supplementary material for: Comprehensive Analysis to Identify Key Genes Involved in Advanced Atherosclerosis
Source: Dis Markers. 2021 Dec 10;2021:4026604. doi: 10.1155/2021/4026604 (PMC8683248; doi:10.1155/2021/4026604)
Supplement: Supplementary Materials — Table S1: the DEGs of GSE28829. Table S2: the DEGs of GSE120521. [file 4026604.f1.zip › Additional file 1.pdf]

TABLE S1: Differentially expressed genes of GSE28829

Gene	logFC	AveExpr	t	P.Value	adj.P.Val	B
VAMP8	2.210496	8.32561	8.167374	7.61E-09	3.84E-05	10.35898
ADAP2	1.984814	6.827842	7.920585	1.39E-08	4.27E-05	9.779878
CTSB	1.586995	11.133	7.648246	2.73E-08	4.58E-05	9.130946
CMTM7	1.106648	5.452838	7.536089	3.61E-08	4.60E-05	8.860746
SLMAP	-1.08599	6.795328	-7.47259	4.23E-08	4.60E-05	8.707026
TMEM35A	-1.94389	5.202206	-7.46267	4.34E-08	4.60E-05	8.682955
LGALS9	1.174631	7.077615	7.332695	6.02E-08	5.11E-05	8.366459
CCR1	2.234003	6.142542	7.321305	6.19E-08	5.11E-05	8.338618
SLAMF8	2.284273	5.640639	7.302293	6.50E-08	5.11E-05	8.292109
CD52	2.555944	5.863032	7.283798	6.81E-08	5.11E-05	8.246823
FCGBP	2.441408	8.156548	7.28194	6.84E-08	5.11E-05	8.24227
ANGPTL1	-1.36596	7.671042	-7.1664	9.18E-08	6.28E-05	7.958332
PARP12	1.127589	6.42595	7.046532	1.25E-07	7.86E-05	7.661967
CD48	1.021248	4.192964	6.98757	1.45E-07	8.61E-05	7.515542
SASH3	1.035629	5.644526	6.951192	1.59E-07	8.93E-05	7.424991
C3AR1	1.998661	8.360221	6.94595	1.62E-07	8.93E-05	7.411929
SELPLG	1.206564	4.59578	6.924988	1.70E-07	8.93E-05	7.359664
AIF1	1.462749	6.416908	6.890194	1.87E-07	8.93E-05	7.272798
NCF4	1.563767	4.408015	6.863384	2.00E-07	8.93E-05	7.205768
PLTP	1.698957	10.24956	6.859995	2.02E-07	8.93E-05	7.197289
GRN	1.139439	9.467811	6.852128	2.06E-07	8.93E-05	7.1776
MYO1F	1.665258	5.644074	6.845064	2.10E-07	8.93E-05	7.159914
AMPD3	1.891281	5.133447	6.828838	2.19E-07	8.93E-05	7.119272
RUBCNL	1.085117	4.132414	6.807687	2.31E-07	8.93E-05	7.066245
BTC	-1.54181	4.222213	-6.77511	2.51E-07	8.93E-05	6.984481
CD14	2.061528	9.464757	6.774228	2.52E-07	8.93E-05	6.982254
KYNU	1.275553	4.086158	6.773366	2.52E-07	8.93E-05	6.980091
MBNL1-AS1	-1.51002	8.44675	-6.75309	2.66E-07	9.10E-05	6.929135
FCGR1B	3.020381	6.329184	6.731153	2.82E-07	9.28E-05	6.873941
RAC2	1.683671	5.768387	6.664205	3.35E-07	0.000106	6.705183
LILRB1	1.244009	6.262131	6.65727	3.41E-07	0.000106	6.687674
ODC1	-1.21824	8.80156	-6.65169	3.46E-07	0.000106	6.673574
FERMT3	1.482695	5.973445	6.598992	3.97E-07	0.000115	6.540325
CD33	1.062821	3.571284	6.543262	4.60E-07	0.000128	6.399076
APOE	1.465368	5.856095	6.540818	4.63E-07	0.000128	6.392872
LINC01094	1.803832	3.942927	6.521132	4.87E-07	0.000129	6.342896
CD83	1.981659	6.082764	6.46277	5.68E-07	0.000145	6.194494
C1QB	2.294893	9.362557	6.452538	5.83E-07	0.000147	6.168438
PTPN6	1.432284	6.864754	6.434474	6.12E-07	0.000152	6.122416
SERPINA1	1.224141	4.832868	6.431643	6.16E-07	0.000152	6.115201
FYB1	1.228107	4.401137	6.427098	6.24E-07	0.000152	6.103614
GIMAP4	1.450064	8.716062	6.411489	6.50E-07	0.000154	6.06381
PIK3AP1	1.676251	4.265094	6.39531	6.78E-07	0.000159	6.022524
ITGB2	2.015598	6.016462	6.328189	8.09E-07	0.000181	5.850977
LAIR1	1.59846	5.326133	6.286715	9.03E-07	0.000199	5.744761
LAPTM5	2.058467	10.28261	6.282109	9.14E-07	0.000199	5.732957
IRF8	1.69422	6.220674	6.2449	1.01E-06	0.000212	5.63751
NPL	1.444821	4.647959	6.230191	1.05E-06	0.000218	5.599746
P2RX7	1.558251	4.080765	6.206527	1.12E-06	0.000226	5.538948

PYCARD	1.384685	6.357614	6.204942	1.12E-06	0.000226	5.534876
HLA-DMB	1.80224	8.386053	6.20217	1.13E-06	0.000226	5.527749
LIPA	1.478885	10.55354	6.198086	1.14E-06	0.000226	5.517251
SLCO2B1	1.265013	5.559717	6.177054	1.21E-06	0.000234	5.463156
SLC7A7	1.235982	7.365916	6.168126	1.24E-06	0.000235	5.440184
NPC2	1.059693	10.77667	6.159685	1.26E-06	0.000238	5.418456
CCL18	3.610852	8.593056	6.114042	1.43E-06	0.00026	5.300865
C4orf48	1.563996	6.556715	6.109575	1.44E-06	0.00026	5.289348
GIMAP1	1.226909	6.090217	6.075518	1.58E-06	0.000272	5.20148
CTSC	1.401321	7.765855	6.040718	1.74E-06	0.000282	5.111597
CORO1A	1.589044	5.980599	6.036786	1.75E-06	0.000283	5.101434
ACP5	3.366351	6.099744	6.034146	1.77E-06	0.000283	5.094612
GIMAP2	1.951243	6.373361	6.03019	1.79E-06	0.000284	5.084385
TMEM176A	1.670404	8.715881	6.020084	1.83E-06	0.000289	5.058258
CD180	1.135099	3.644515	6.011403	1.88E-06	0.00029	5.035807
MARCHF1	1.205706	4.111849	5.993866	1.97E-06	0.000293	4.990435
HCK	2.148379	6.215207	5.985423	2.01E-06	0.000293	4.968581
CRYBG1	1.863413	6.045562	5.94613	2.23E-06	0.000321	4.866813
SLC15A3	1.486765	5.998505	5.941524	2.26E-06	0.000321	4.854875
SIGLEC1	1.640951	5.706343	5.919451	2.40E-06	0.000329	4.797648
NIPSNAP3E	-1.4426	4.732216	-5.89226	2.58E-06	0.000345	4.727101
ACADL	-1.98169	6.204505	-5.85467	2.85E-06	0.000369	4.629509
CPVL	1.866974	7.009104	5.824323	3.10E-06	0.000383	4.550626
LINC01503	1.008096	5.08053	5.823227	3.10E-06	0.000383	4.547777
C3orf70	-1.23052	6.435877	-5.81043	3.21E-06	0.000388	4.514497
LY86	2.380754	5.977487	5.793649	3.36E-06	0.000401	4.470844
CCL19	2.7715	7.957774	5.778529	3.50E-06	0.000413	4.431496
CSF1R	1.845627	7.393541	5.736084	3.92E-06	0.000445	4.320963
HLA-DMA	1.815925	8.327273	5.733553	3.95E-06	0.000445	4.314367
ARL4A	1.302002	8.539545	5.723996	4.05E-06	0.000454	4.289462
TYROBP	2.097178	8.201709	5.721692	4.08E-06	0.000455	4.283458
C1QC	2.078839	9.477638	5.710549	4.20E-06	0.000463	4.254412
APOC1	2.498297	7.375357	5.700039	4.32E-06	0.000469	4.227011
LHFPL2	1.068317	7.953958	5.681613	4.54E-06	0.000485	4.178954
CD37	1.91386	4.925922	5.678631	4.58E-06	0.000485	4.171177
GPX1	1.04606	10.55397	5.677623	4.59E-06	0.000485	4.168546
CASP1	1.349126	6.293681	5.677257	4.60E-06	0.000485	4.167591
OSBPL3	1.091882	3.366787	5.671957	4.66E-06	0.00049	4.153765
CCL4	1.775853	5.861094	5.669193	4.70E-06	0.00049	4.146552
STEAP1	1.154138	7.746488	5.667952	4.71E-06	0.00049	4.143314
MARCO	1.832528	6.914361	5.627009	5.26E-06	0.000531	4.036437
ATP1A2	-2.08843	7.805465	-5.60148	5.64E-06	0.000557	3.96976
MAFB	1.298508	9.258448	5.594806	5.74E-06	0.000562	3.952315
TLR2	1.372166	7.347577	5.568407	6.16E-06	0.000586	3.883317
NCKAP1L	1.089688	5.008175	5.53097	6.82E-06	0.000628	3.785414
TNFSF13B	2.654474	7.250404	5.512964	7.16E-06	0.000646	3.738302
ACTN2	-1.02927	3.906718	-5.49392	7.54E-06	0.000654	3.68847
CNTN4	-1.44878	7.030512	-5.4927	7.56E-06	0.000654	3.685272
SH3BGR	-1.51864	7.833665	-5.49201	7.57E-06	0.000654	3.683465
C1QA	1.819823	9.542699	5.489785	7.62E-06	0.000654	3.677638
IGLL3P	2.817129	7.580864	5.483929	7.74E-06	0.000658	3.662308

IER3	2.41089	8.816547	5.477244	7.88E-06	0.000665	3.644809
LOC100287	-1.2031	5.069557	-5.47538	7.92E-06	0.000666	3.639927
FABP5	1.922415	8.773134	5.467052	8.10E-06	0.000672	3.618122
FCGR2C	1.109872	5.147263	5.451154	8.46E-06	0.000694	3.576488
SPP1	3.191765	8.11448	5.448029	8.53E-06	0.000697	3.568303
PLEK	1.605843	6.478745	5.438908	8.74E-06	0.000711	3.544411
GMFG	1.607211	6.073956	5.434367	8.85E-06	0.000715	3.532516
CTSS	2.299836	7.248959	5.433771	8.87E-06	0.000715	3.530953
SNX10	1.787588	6.592914	5.431673	8.92E-06	0.000717	3.525458
HK3	1.308625	3.996373	5.427049	9.03E-06	0.000723	3.513344
FBP1	2.343335	4.472319	5.422031	9.15E-06	0.000729	3.500194
C14orf132	-1.00974	6.76572	-5.42106	9.18E-06	0.000729	3.497664
MARCKS	1.32907	8.316121	5.394018	9.87E-06	0.00076	3.426785
TNFRSF1B	1.362843	6.82248	5.37048	1.05E-05	0.000786	3.36508
TREM2	2.021411	5.02383	5.323024	1.20E-05	0.000875	3.240632
VSIG4	1.788756	9.454051	5.320374	1.21E-05	0.000878	3.233681
PLA2G2A	1.226391	12.5325	5.317218	1.22E-05	0.00088	3.225403
HLA-DRA	1.69121	11.13756	5.315611	1.22E-05	0.00088	3.221186
DNAJB5	-1.06176	4.83137	-5.31134	1.24E-05	0.000883	3.209974
JCHAIN	4.206317	7.194092	5.311051	1.24E-05	0.000883	3.209226
CCRL2	1.333208	4.515363	5.303216	1.26E-05	0.000894	3.188671
NCF2	2.127591	5.666671	5.279994	1.34E-05	0.000939	3.127741
FAM167B	1.006856	3.621894	5.277928	1.35E-05	0.000941	3.122321
MS4A6A	1.553251	6.099787	5.27519	1.36E-05	0.000944	3.115135
LST1	1.036561	5.29224	5.266556	1.39E-05	0.000957	3.092477
IGFBP4	1.046004	10.67494	5.264401	1.40E-05	0.000959	3.086824
FCGR2B	2.86531	6.570006	5.259883	1.42E-05	0.000968	3.074967
ADAMDEC1	2.437171	4.024996	5.223407	1.57E-05	0.001044	2.979226
CSF2RB	2.49864	5.631638	5.211891	1.62E-05	0.001056	2.948996
TIAM1	1.047565	3.975514	5.209719	1.63E-05	0.001059	2.943294
PLA2G7	1.750914	4.535508	5.194182	1.70E-05	0.001094	2.902507
IL2RG	1.432845	5.299438	5.191207	1.71E-05	0.001096	2.894696
CD93	1.16788	8.656789	5.179813	1.76E-05	0.001109	2.864781
BCAT1	1.342633	6.22593	5.16781	1.82E-05	0.001125	2.833266
PTGS1	1.166122	5.461717	5.165378	1.83E-05	0.001125	2.82688
SBSPON	-1.18129	6.100555	-5.15749	1.87E-05	0.001133	2.806164
CKB	-1.04044	7.612144	-5.11935	2.08E-05	0.001191	2.706017
PAG1	1.009075	5.205217	5.102816	2.17E-05	0.001236	2.662598
CTSD	1.069369	8.062184	5.077571	2.33E-05	0.001287	2.596302
LONRF2	-1.02269	3.788928	-5.06004	2.44E-05	0.001339	2.550258
LY96	1.660446	7.292301	5.046572	2.53E-05	0.00137	2.514896
ARL4C	1.413381	5.574075	5.03206	2.63E-05	0.001414	2.47679
ZWINT	1.508822	4.153835	4.99493	2.91E-05	0.001512	2.379292
MS4A4A	2.096733	7.408611	4.956281	3.24E-05	0.001629	2.27782
LCP1	1.682654	7.730775	4.941553	3.37E-05	0.001666	2.239157
NEXN	-1.13068	10.37732	-4.93754	3.41E-05	0.001676	2.22862
G0S2	1.191989	6.421879	4.899842	3.77E-05	0.001788	2.129682
CXCL16	1.887856	7.287969	4.89384	3.84E-05	0.001807	2.11393
ABCG1	1.009385	4.898036	4.878755	4.00E-05	0.001853	2.074349
LGMN	1.130802	9.076455	4.876607	4.02E-05	0.001853	2.068712
GALNT6	1.035754	2.949745	4.875234	4.03E-05	0.001853	2.06511

RNASE6	1.964572	6.914452	4.873705	4.05E-05	0.001853	2.061098
CHI3L1	1.682064	5.618512	4.861404	4.19E-05	0.001906	2.028826
FCER1G	1.99546	8.517212	4.85417	4.27E-05	0.001928	2.00985
ITGAM	1.018619	4.759627	4.849768	4.32E-05	0.001943	1.998301
LSP1	1.306031	7.401758	4.842778	4.41E-05	0.001962	1.979968
TLR1	1.452148	4.297488	4.840901	4.43E-05	0.001968	1.975045
IL10RA	1.600064	6.279423	4.838655	4.46E-05	0.001971	1.969153
UCP2	1.125783	6.962026	4.803588	4.90E-05	0.002077	1.8772
CLEC5A	1.935108	5.398195	4.802541	4.92E-05	0.002077	1.874455
TREM1	1.918719	6.260147	4.789651	5.09E-05	0.002108	1.840665
PTTG1	1.15634	5.243423	4.783887	5.17E-05	0.00212	1.825557
CP	1.068291	8.553055	4.773109	5.32E-05	0.002155	1.797307
PDZRN4	-1.13724	6.128518	-4.76223	5.48E-05	0.002204	1.768797
TLCD4	-1.27641	5.549337	-4.74913	5.68E-05	0.002261	1.734477
ZNRF3	-1.03606	6.9627	-4.73473	5.91E-05	0.002301	1.696759
CXCR4	2.386761	8.629282	4.716769	6.20E-05	0.002379	1.649721
MS4A7	1.722068	6.676637	4.681904	6.82E-05	0.002558	1.558458
CAPG	2.155115	7.337824	4.666189	7.12E-05	0.002625	1.517342
HCLS1	1.320439	8.061085	4.666112	7.12E-05	0.002625	1.51714
GIMAP6	1.783369	7.123987	4.655872	7.32E-05	0.002675	1.490354
COLEC12	1.511791	9.043703	4.655848	7.32E-05	0.002675	1.49029
APCDD1	-1.301	7.118549	-4.63978	7.65E-05	0.002749	1.44826
C15orf48	2.145698	3.729296	4.638287	7.68E-05	0.002751	1.444367
RAB23	-1.08586	8.289331	-4.63602	7.72E-05	0.002753	1.438449
PLAUR	1.365938	7.115853	4.631466	7.82E-05	0.002777	1.426535
TRBC1	1.543217	5.618736	4.630241	7.85E-05	0.002782	1.423333
DRAM1	1.002539	8.303491	4.615491	8.17E-05	0.00286	1.384782
LILRB2	1.029423	4.706206	4.612538	8.23E-05	0.00286	1.377064
CD74	1.063513	7.301368	4.612327	8.24E-05	0.00286	1.376512
CKS2	1.444187	3.818785	4.610025	8.29E-05	0.002863	1.370496
LYN	1.270762	7.264065	4.60658	8.37E-05	0.00288	1.361494
HHEX	1.222303	5.659795	4.596153	8.61E-05	0.002931	1.334254
TLR5	1.183518	5.719273	4.595912	8.61E-05	0.002931	1.333626
MPEG1	1.834813	6.868225	4.571998	9.19E-05	0.003024	1.271177
GPR65	1.048281	3.825503	4.548038	9.80E-05	0.00317	1.208641
PTPRC	1.253813	5.014666	4.533522	0.000102	0.003276	1.170775
ALOX5AP	1.72663	8.313508	4.52954	0.000103	0.003301	1.16039
CD86	1.610338	4.593382	4.525864	0.000104	0.003318	1.150803
CEMIP	1.016469	3.464869	4.501449	0.000111	0.003447	1.08716
CXCL12	1.096784	9.704181	4.49976	0.000112	0.003457	1.08276
TNFAIP3	1.459889	8.463206	4.478607	0.000118	0.003606	1.027655
ARRB2	1.347185	5.923175	4.474269	0.00012	0.003632	1.016361
PCDHB16	-1.42273	6.579387	-4.47084	0.000121	0.00366	1.007427
ECM1	1.151613	7.537659	4.461437	0.000124	0.003743	0.982953
PTRHD1	1.000443	6.485034	4.449764	0.000128	0.003843	0.952576
CTPS1	-1.02423	7.42634	-4.44265	0.00013	0.003903	0.934064
BAMBI	-1.31566	6.667262	-4.42652	0.000136	0.00403	0.892128
EVI2B	2.190072	7.252087	4.40745	0.000143	0.004206	0.842556
MSR1	1.104374	4.024078	4.403586	0.000145	0.004225	0.832516
CCL5	1.5385	6.155503	4.368065	0.00016	0.004539	0.74029
RGS17	-1.01751	4.279213	-4.35838	0.000164	0.004607	0.715167

RRM2	1.563508	3.478309	4.305674	0.000189	0.00507	0.578591
GIMAP7	1.350909	8.107742	4.291821	0.000196	0.005202	0.542741
MLPH	1.076185	5.879216	4.276632	0.000204	0.00535	0.503458
AMIGO2	-1.07205	9.714766	-4.26815	0.000209	0.005409	0.481544
EDN1	1.176497	6.322217	4.243804	0.000223	0.005702	0.418639
ZNF521	1.276914	6.943361	4.228748	0.000232	0.005886	0.379781
MMP9	3.066808	6.494542	4.227581	0.000233	0.005897	0.37677
UGCG	1.000936	8.408031	4.214437	0.000241	0.006012	0.342868
CNTN1	-1.1195	4.337932	-4.20172	0.00025	0.00617	0.310078
CHI3L2	2.330026	4.832043	4.200613	0.00025	0.00617	0.307236
PLD5	-1.25849	4.427353	-4.19058	0.000257	0.006269	0.281394
REEP1	-1.30674	7.521223	-4.18593	0.000261	0.00631	0.269402
GPR137B	1.011803	7.833204	4.166846	0.000274	0.006524	0.220294
FPR3	1.680078	4.974381	4.164595	0.000276	0.006549	0.214503
SLA	1.433959	7.262488	4.160601	0.000279	0.006604	0.204232
EVI2A	1.821957	6.657879	4.154772	0.000283	0.00668	0.189242
SGK1	1.438968	8.31318	4.134345	0.000299	0.006931	0.136751
IGLC1	1.084828	3.974193	4.127105	0.000305	0.007018	0.118157
CD3D	1.030392	5.226409	4.116178	0.000314	0.007139	0.090111
JAML	1.17495	5.488086	4.115281	0.000315	0.007147	0.087809
HLA-DPA1	1.510425	9.522929	4.112704	0.000317	0.007149	0.081197
C7	1.119595	8.837409	4.111391	0.000318	0.007149	0.077829
CCL8	1.863484	5.608158	4.10832	0.000321	0.007162	0.069952
TRAC	1.211762	5.287364	4.100873	0.000327	0.007257	0.050856
PLCXD3	1.393609	6.688028	4.095337	0.000332	0.007309	0.036662
CSTA	1.421878	6.068398	4.090829	0.000336	0.007358	0.025111
RCSD1	1.083783	5.401681	4.087006	0.00034	0.007417	0.015314
IL32	1.24214	6.505848	4.077768	0.000348	0.00757	-0.00835
CD72	1.540816	4.403365	4.071759	0.000354	0.007643	-0.02373
CLSTN2	1.189368	5.88428	4.070333	0.000355	0.007656	-0.02738
PLAU	1.104195	6.979776	4.069898	0.000356	0.007657	-0.02849
INPP5A	-1.14098	7.554995	-4.03611	0.000389	0.008112	-0.11489
ADRA2A	1.656124	6.039711	4.020505	0.000406	0.008399	-0.15474
HCP5	1.016332	4.193357	4.01766	0.000409	0.008443	-0.162
TCEAL2	-1.02834	8.999418	-4.01242	0.000415	0.008513	-0.17537
LGR6	-1.51269	7.693812	-4.00927	0.000418	0.008529	-0.18341
TOX2	-1.21254	4.055171	-4.00853	0.000419	0.008529	-0.1853
PRUNE2	-1.01986	8.760816	-4.0041	0.000424	0.008605	-0.1966
CNN1	-1.2354	11.88176	-3.99863	0.00043	0.008703	-0.21054
CYP1B1	1.369744	7.285598	3.991134	0.000439	0.008778	-0.22963
THY1	1.363	6.682153	3.983146	0.000448	0.00892	-0.24998
NEURL1B	1.213532	7.242161	3.979667	0.000453	0.008985	-0.25884
KCNT2	1.652418	7.044907	3.958878	0.000478	0.009395	-0.31173
THEMIS2	1.532349	5.068663	3.949764	0.00049	0.00957	-0.33489
LXN	1.214441	8.074982	3.93977	0.000503	0.009715	-0.36028
MXRA5	1.770602	6.848533	3.935995	0.000508	0.009803	-0.36986
TNFRSF21	1.174109	4.938637	3.91971	0.000531	0.010121	-0.41118
IL7R	1.483038	4.035115	3.91828	0.000533	0.01015	-0.41481
FXYD6	1.03939	6.141154	3.913665	0.000539	0.010256	-0.42651
SECTM1	1.255037	5.091607	3.897277	0.000563	0.010623	-0.46803
CHMP4C	-1.59197	4.896462	-3.89515	0.000567	0.010651	-0.4734

ADORA3	1.363403	4.214216	3.892465	0.000571	0.010659	-0.48021
CFI	1.706349	7.347497	3.891099	0.000573	0.010688	-0.48367
HS3ST2	1.448351	7.192648	3.889085	0.000576	0.010735	-0.48876
ADGRF5	1.029287	8.140273	3.875525	0.000597	0.010986	-0.52306
EPB41L3	1.49623	7.039476	3.874928	0.000598	0.010994	-0.52457
SLC18B1	1.51743	6.109575	3.864918	0.000614	0.01118	-0.54987
SYNC	-1.25509	7.664881	-3.85175	0.000636	0.011392	-0.58312
OPN3	1.087314	5.570729	3.836255	0.000662	0.011782	-0.6222
FIBIN	-1.02282	9.587938	-3.81747	0.000696	0.0122	-0.66952
GAS2L3	1.201012	5.157679	3.80895	0.000712	0.0124	-0.69097
BCL2A1	2.05103	5.743968	3.791248	0.000746	0.012786	-0.73547
AQP9	2.098054	5.72088	3.784969	0.000758	0.012948	-0.75124
PLBD1	1.096176	8.110786	3.778344	0.000772	0.013139	-0.76787
NEURL2	1.035357	3.450826	3.753848	0.000823	0.013829	-0.82929
CSPG4	-1.13688	6.54949	-3.75053	0.000831	0.013927	-0.83761
ACKR1	1.175973	10.31009	3.747359	0.000837	0.014009	-0.84554
ARHGEF3	1.105127	8.209888	3.734682	0.000866	0.014307	-0.87726
GZMK	1.418542	4.436217	3.724713	0.000889	0.014556	-0.90218
PNMA8A	-1.52427	7.308222	-3.72039	0.000899	0.014651	-0.91299
KCNK17	-1.58741	7.342051	-3.70829	0.000928	0.015003	-0.94318
ARHGAP18	1.114664	5.823426	3.6661	0.001037	0.016226	-1.04827
IFI27	1.074206	8.083909	3.654438	0.001069	0.016588	-1.07725
HILPDA	1.170044	9.39191	3.654417	0.001069	0.016588	-1.0773
IGKC	1.243352	3.939447	3.646321	0.001092	0.016853	-1.0974
IGSF21	1.087767	4.096706	3.632805	0.001131	0.017319	-1.13092
FGR	1.496195	5.929891	3.628319	0.001144	0.017451	-1.14204
MYOZ1	-1.0718	6.000784	-3.61603	0.001182	0.01781	-1.17248
BLNK	1.567389	4.554146	3.615546	0.001183	0.017815	-1.17367
VCAM1	1.598346	9.072499	3.611212	0.001197	0.017928	-1.18439
ZNF267	1.04072	5.888808	3.597307	0.001241	0.018376	-1.21876
GPR34	1.877239	6.029764	3.553486	0.001391	0.019873	-1.32679
FLNC	-1.21531	7.357798	-3.52807	0.001486	0.020875	-1.38921
GSTT2	-1.15636	3.216393	-3.52701	0.00149	0.020903	-1.39182
IGHM	2.141202	4.503695	3.523292	0.001504	0.021047	-1.40094
MCUB	1.229162	7.039876	3.513439	0.001543	0.021517	-1.42509
RAMP1	-1.15178	8.667867	-3.49105	0.001635	0.022418	-1.47987
CYTIP	1.862764	6.821826	3.480816	0.001679	0.022784	-1.50487
LMOD1	-1.01005	10.51616	-3.47989	0.001683	0.022807	-1.50712
F13A1	1.746456	9.393483	3.472736	0.001715	0.023142	-1.52459
ALDH1B1	-1.09682	7.278898	-3.47244	0.001716	0.023142	-1.52532
C1orf162	1.07588	8.116747	3.44553	0.00184	0.024287	-1.59085
FCGR3B	1.246491	5.605321	3.435801	0.001886	0.02463	-1.6145
GZMA	1.242365	4.024724	3.423714	0.001946	0.025246	-1.64384
PSMB9	1.124889	6.325572	3.398318	0.002077	0.026468	-1.70536
CPM	1.020876	4.797344	3.384119	0.002154	0.027052	-1.73968
TPH1	-1.44674	5.242385	-3.384	0.002155	0.027052	-1.73996
RAMP3	1.115252	7.068302	3.382872	0.002161	0.027092	-1.74269
AMPH	-1.01392	4.64416	-3.3823	0.002164	0.027103	-1.74408
RGS1	1.248249	6.35256	3.369229	0.002238	0.027692	-1.7756
HMOX1	1.613497	5.71716	3.331608	0.002464	0.029519	-1.86609
LUM	1.287857	9.339099	3.323357	0.002516	0.029951	-1.88587

C3	1.018306	12.40436	3.312182	0.002589	0.030476	-1.91264
MRC1	1.493768	8.709181	3.286509	0.002764	0.032103	-1.974
S100A9	1.74411	8.493279	3.284297	0.00278	0.032228	-1.97928
SMAGP	1.021467	4.344502	3.274959	0.002846	0.032776	-2.00154
CXCL14	1.289811	7.008824	3.256322	0.002984	0.033843	-2.04588
HP	2.180635	6.927501	3.245934	0.003064	0.034409	-2.07056
SLC16A10	1.328634	3.605333	3.240063	0.00311	0.034717	-2.08448
MMP12	3.285332	4.691748	3.23985	0.003111	0.034717	-2.08499
LINC00702	-1.07492	7.829763	-3.23741	0.003131	0.034856	-2.09078
EMCN	1.116578	7.883851	3.157647	0.003828	0.040366	-2.27882
MYO5C	1.053758	6.155085	3.154412	0.003859	0.040652	-2.28641
CASQ2	-1.4559	8.293151	-3.13893	0.004012	0.041846	-2.32264
MS4A14	1.05685	5.559194	3.128681	0.004116	0.042559	-2.34659
GPR183	1.568482	7.23685	3.127565	0.004127	0.042635	-2.34919
CCDC146	-1.42729	7.460361	-3.11202	0.004291	0.043987	-2.38544
POU2AF1	1.671772	3.627618	3.100869	0.004412	0.044638	-2.41138
SMAP2	1.01341	6.206595	3.099911	0.004423	0.044722	-2.41361
ST6GAL2	-1.2589	6.929639	-3.09491	0.004478	0.045125	-2.42522
C5AR1	1.601028	7.486487	3.085021	0.00459	0.045777	-2.44818
TM4SF18	1.145809	5.24862	3.082782	0.004615	0.045922	-2.45337
HPGDS	1.268253	5.085244	3.068006	0.004788	0.047172	-2.48758
SMPX	-1.15335	3.116857	-3.06796	0.004789	0.047172	-2.48769
MYOCD	-1.24148	9.188652	-3.0578	0.004911	0.048049	-2.51117
NOSTRIN	1.002218	6.64847	3.040882	0.005122	0.049507	-2.55019
TM6SF1	1.03481	5.998504	3.039739	0.005136	0.049624	-2.55282

Abbreviations: FC: fold change; AveExpr: average expression; adj.P.Val: adjust P value
