# Supplementary material for: Comprehensive Analysis to Identify Key Genes Involved in Advanced Atherosclerosis
Source: Dis Markers. 2021 Dec 10;2021:4026604. doi: 10.1155/2021/4026604 (PMC8683248; doi:10.1155/2021/4026604)
Supplement: Supplementary Materials — Table S1: the DEGs of GSE28829. Table S2: the DEGs of GSE120521. [file 4026604.f1.zip › Additional file 2.pdf]

TABLE S2: Differentially expressed genes of GSE120521

Gene	logFC	AveExpr	t	P.Value	adj.P.Val	B
TM4SF19	2.828539	4.034474	13.53259	8.91E-07	0.004594	6.117689
SLC24A3	-2.32322	3.52143	-12.9666	1.24E-06	0.004594	5.857048
HIST1H1B	2.023915	5.933349	12.91923	1.27E-06	0.004594	5.83447
HRCT1	-1.86711	3.177071	-12.8991	1.29E-06	0.004594	5.824848
ALOX5AP	1.741036	6.221504	12.03993	2.17E-06	0.005586	5.393029
IGFBPL1	-3.08158	2.855753	-11.7591	2.60E-06	0.005586	5.242502
HIST1H3I	1.757573	5.111236	11.42574	3.23E-06	0.005586	5.057375
VNN1	1.57264	3.676654	11.05379	4.15E-06	0.005586	4.842183
ADRA1B	-2.74192	2.152516	-10.9249	4.53E-06	0.005586	4.76537
ATP6V0D2	2.584503	2.369012	10.91153	4.57E-06	0.005586	4.757367
SGCA	-1.7697	6.442156	-10.8832	4.66E-06	0.005586	4.740333
WFDC1	-2.79036	4.513453	-10.8736	4.69E-06	0.005586	4.734494
MEGF6	-1.89233	6.627743	-10.2975	7.05E-06	0.006114	4.374478
CSDC2	-1.92593	3.429077	-10.2805	7.14E-06	0.006114	4.363416
TNC	2.166311	8.077772	10.2617	7.24E-06	0.006114	4.351247
MMP7	4.912725	3.510353	10.26099	7.24E-06	0.006114	4.350786
HIST1H2BH	1.356128	3.274947	10.25473	7.28E-06	0.006114	4.34672
EFEMP1	-1.28465	10.54626	-9.83729	9.91E-06	0.006863	4.068429
NGF	-1.91314	2.760764	-9.78457	1.03E-05	0.006863	4.032267
PTGIS	-1.31961	7.617775	-9.75117	1.06E-05	0.006863	4.009245
RIPK4	-2.06063	2.391248	-9.6421	1.15E-05	0.006863	3.933388
DUSP26	-2.76495	3.795255	-9.62619	1.16E-05	0.006863	3.922236
SVEP1	2.012893	4.210926	9.582313	1.20E-05	0.006863	3.891378
PPP1R14A	-2.91338	6.740267	-9.54792	1.24E-05	0.006863	3.867069
ANO1	-1.86972	5.755015	-9.5384	1.25E-05	0.006863	3.860325
MGARP	-1.91893	2.892894	-9.49256	1.29E-05	0.006863	3.827731
CACNA1H	-1.9684	3.089194	-9.48596	1.30E-05	0.006863	3.823021
MASP1	-3.62851	3.801639	-9.40987	1.38E-05	0.007022	3.768471
CITED4	-1.65503	3.458613	-9.29281	1.51E-05	0.007232	3.683539
NPR1	-2.8047	4.535352	-9.21045	1.61E-05	0.007232	3.623042
CCDC190	-2.35759	2.499604	-9.20613	1.62E-05	0.007232	3.619853
HIST1H3B	2.308467	6.510356	9.203801	1.62E-05	0.007232	3.618134
GAS6	-1.94677	8.954461	-9.05163	1.83E-05	0.007311	3.504633
HPGD	2.614429	3.441418	9.045971	1.84E-05	0.007311	3.500371
GRK5	-1.572	4.842368	-9.03116	1.86E-05	0.007311	3.489199
FBLN5	-2.17539	7.238723	-8.98732	1.93E-05	0.007311	3.45602
TNFSF15	1.229193	2.063205	8.985692	1.93E-05	0.007311	3.454782
IBSP	4.759103	4.763951	8.977221	1.95E-05	0.007311	3.448348
MTUS2	-2.25753	2.524787	-8.8071	2.24E-05	0.008186	3.317688
MPPED2	-2.27023	3.347095	-8.75046	2.34E-05	0.008186	3.273563
CLEC18A	-2.6359	2.959273	-8.69266	2.46E-05	0.008186	3.22821
SLC25A4	-2.0633	6.245816	-8.64731	2.56E-05	0.008186	3.192398
HIST2H3C	1.705754	4.387277	8.641602	2.57E-05	0.008186	3.187875
HIST2H3A	1.705754	4.387277	8.641602	2.57E-05	0.008186	3.187875
KRT86	-1.66202	1.991469	-8.61888	2.62E-05	0.008186	3.16984
PLBD1	2.080515	5.362418	8.585594	2.69E-05	0.008186	3.14333
GPR146	1.836579	2.483634	8.539397	2.80E-05	0.008186	3.106351
HIST1H3C	1.735492	5.840878	8.486243	2.93E-05	0.008186	3.063535
NHS	-1.73054	3.991443	-8.46554	2.98E-05	0.008186	3.046785

IL17D	-1.2538	1.914179	-8.45825	3.00E-05	0.008186	3.040874
TMEM47	-2.33005	6.588171	-8.45466	3.01E-05	0.008186	3.037956
MICAL2	-1.12173	8.165941	-8.45209	3.02E-05	0.008186	3.035877
SLC14A1	-2.60607	6.494206	-8.44409	3.04E-05	0.008186	3.029378
ST18	2.129366	1.994987	8.401013	3.15E-05	0.008338	2.994281
LIPA	1.959311	7.729077	8.348079	3.30E-05	0.008444	2.950891
FBXO32	-1.72259	6.746718	-8.34427	3.31E-05	0.008444	2.947755
GPAT3	1.435344	3.281029	8.248128	3.60E-05	0.008875	2.868163
CENPF	1.361093	2.13406	8.241598	3.62E-05	0.008875	2.862722
SLC26A10	-1.6066	2.928132	-8.2096	3.72E-05	0.008875	2.835993
DCSTAMP	2.458185	2.33922	8.207986	3.73E-05	0.008875	2.834642
FGF1	-2.30007	5.061431	-8.16084	3.89E-05	0.008949	2.79505
PKDCC	-2.06436	5.511269	-8.16011	3.89E-05	0.008949	2.79444
HIST1H1D	1.206637	6.291274	8.139313	3.96E-05	0.008949	2.776897
ATOH8	-2.49103	4.660687	-8.08783	4.15E-05	0.008949	2.733272
DMPK	-1.59751	5.98066	-8.08148	4.17E-05	0.008949	2.727875
HIST1H2AM	1.638483	5.938776	8.081208	4.17E-05	0.008949	2.727642
CCNB1	1.434104	3.189857	8.073645	4.20E-05	0.008949	2.721204
PKIG	-1.07877	6.581872	-8.05004	4.29E-05	0.009005	2.701069
KLHL30	-1.87884	3.141371	-8.03062	4.36E-05	0.00903	2.684462
SLC7A8	1.874317	4.650812	7.954418	4.67E-05	0.009412	2.618887
GALNT18	-1.75664	4.558676	-7.9472	4.70E-05	0.009412	2.61264
TPM2	-2.57254	9.799345	-7.93295	4.76E-05	0.009412	2.600298
MYO18B	-2.21887	2.140344	-7.92179	4.81E-05	0.009412	2.59061
DNAH14	1.226948	2.512182	7.87742	5.01E-05	0.009476	2.551975
FIBIN	-2.37657	6.298184	-7.84251	5.17E-05	0.009476	2.521418
LYVE1	1.75732	3.863704	7.841657	5.17E-05	0.009476	2.520672
EFS	-1.17875	3.423551	-7.83763	5.19E-05	0.009476	2.51714
VIPR2	-2.40922	2.436532	-7.83243	5.22E-05	0.009476	2.512576
NALCN	-2.29627	3.505367	-7.82725	5.24E-05	0.009476	2.508019
PRKAA2	-1.6333	2.939607	-7.81215	5.31E-05	0.009487	2.494739
GREM1	4.078374	2.913308	7.776345	5.49E-05	0.009561	2.463131
CD33	1.867765	4.331165	7.764071	5.55E-05	0.009561	2.452264
GMPR	-1.64853	3.580388	-7.7635	5.56E-05	0.009561	2.45176
ANK3	-1.4161	5.934193	-7.72761	5.74E-05	0.009764	2.419882
CTC-487M2	-1.10698	1.323654	-7.69908	5.89E-05	0.009883	2.394433
GOLGA7B	1.238967	1.592054	7.688975	5.95E-05	0.009883	2.385399
BLID	-1.26923	3.762668	-7.67089	6.05E-05	0.009934	2.369203
EHD3	-1.3681	4.617947	-7.63073	6.28E-05	0.010089	2.333087
TAGLN	-2.11346	12.20477	-7.62981	6.29E-05	0.010089	2.332264
AOC3	-2.35247	5.978948	-7.5898	6.52E-05	0.010298	2.2961
LMNB1	1.102217	3.783452	7.580327	6.58E-05	0.010298	2.287507
ZWINT	1.12422	3.163543	7.532234	6.89E-05	0.010298	2.243736
HIST1H3F	2.004323	6.172177	7.511556	7.02E-05	0.010298	2.224832
ZMIZ1	1.064718	4.637122	7.497728	7.11E-05	0.010298	2.212162
TNFRSF11A	1.736873	2.746445	7.487912	7.18E-05	0.010298	2.203155
NTF3	-2.36573	3.335108	-7.48022	7.23E-05	0.010298	2.19609
LPXN	1.219684	5.161415	7.471395	7.29E-05	0.010298	2.187973
FAM167B	1.401231	1.683426	7.44772	7.46E-05	0.010298	2.166154
CNKSR3	-1.26199	4.197985	-7.44766	7.46E-05	0.010298	2.166098
HMCN1	-1.89398	7.076534	-7.44284	7.49E-05	0.010298	2.161652

DMD	-1.72847	6.665929	-7.43287	7.56E-05	0.010298	2.152431
SHROOM3	-2.36528	4.917777	-7.4227	7.64E-05	0.010298	2.143023
SLC30A3	-2.1464	1.898567	-7.41929	7.66E-05	0.010298	2.139868
MYEF2	-2.12178	5.342043	-7.41015	7.73E-05	0.010298	2.131395
ANGPTL1	-2.3204	3.413262	-7.40718	7.75E-05	0.010298	2.128637
TPM1	-1.53332	9.889913	-7.38892	7.89E-05	0.010298	2.111675
ACSL5	1.228648	4.190564	7.387362	7.90E-05	0.010298	2.110224
KCNA5	-3.39327	2.869115	-7.35874	8.12E-05	0.010298	2.083546
MXRA7	-1.11295	7.524827	-7.35546	8.14E-05	0.010298	2.080489
HIST2H4A	1.187753	7.734148	7.349957	8.19E-05	0.010298	2.075344
NSUN7	-2.0079	2.46322	-7.34674	8.21E-05	0.010298	2.072334
CCRL2	1.726957	3.326305	7.343731	8.24E-05	0.010298	2.069522
PEAR1	-1.19893	3.729323	-7.33135	8.33E-05	0.010298	2.057929
ARHGEF25	-1.57471	5.288116	-7.32973	8.35E-05	0.010298	2.056414
INHBA	-1.65227	6.013137	-7.32778	8.36E-05	0.010298	2.054583
MFGE8	-1.31889	10.06456	-7.29867	8.60E-05	0.010344	2.027241
CPXM2	-1.53024	6.887486	-7.2945	8.63E-05	0.010344	2.023319
TNFRSF12A	-1.28216	7.06765	-7.28012	8.76E-05	0.010344	2.009769
FSTL1	-1.00691	9.461097	-7.26854	8.85E-05	0.010344	1.998833
ATP8B2	-1.07378	6.335216	-7.25325	8.99E-05	0.010344	1.984376
TRPC4	-1.70505	1.933248	-7.24995	9.01E-05	0.010344	1.981253
FOXL1	-1.47878	4.135562	-7.24451	9.06E-05	0.010344	1.9761
HIST1H3J	2.212954	4.541767	7.222871	9.25E-05	0.010344	1.955562
F11R	1.506565	4.420777	7.217578	9.30E-05	0.010344	1.950531
HTR4	-1.92628	2.172356	-7.20517	9.41E-05	0.010344	1.938721
JPH2	-2.69559	4.297212	-7.18945	9.56E-05	0.010344	1.923736
TJP2	-1.03298	5.855577	-7.1706	9.74E-05	0.010344	1.905714
GATA6	-1.25575	4.924088	-7.16805	9.76E-05	0.010344	1.903279
SMARCD3	-1.18467	5.730606	-7.16541	9.79E-05	0.010344	1.90075
COLEC12	1.303432	5.200862	7.165362	9.79E-05	0.010344	1.900706
CCDC102A	-1.00016	4.010127	-7.16174	9.82E-05	0.010344	1.897239
FANCI	1.285229	3.058411	7.152566	9.91E-05	0.010344	1.888444
GLA	1.289303	5.447972	7.146648	9.97E-05	0.010344	1.882766
ITIH5	-2.28654	4.474933	-7.14624	9.97E-05	0.010344	1.882373
MMP12	4.941053	4.807419	7.144045	9.99E-05	0.010344	1.880267
CD151	-1.15587	9.009707	-7.12191	0.000102	0.010429	1.858989
RAMP1	-2.65043	6.819766	-7.10447	0.000104	0.010432	1.842172
FADS2	-1.14353	5.942676	-7.09908	0.000104	0.010432	1.836968
PTPN22	1.698689	4.18049	7.07604	0.000107	0.010546	1.814684
SH2D3C	1.294248	3.071527	7.060343	0.000109	0.010546	1.799464
MYH10	-2.80812	8.924467	-7.04659	0.00011	0.010546	1.786104
RYR3	-1.21995	2.681588	-7.03608	0.000111	0.010546	1.775877
CYTL1	-3.05315	5.302945	-7.03517	0.000111	0.010546	1.774992
NXPH3	-2.27338	4.687327	-7.02836	0.000112	0.010546	1.768357
TDO2	3.503849	3.376432	7.023926	0.000112	0.010546	1.764033
TCEAL3	-1.0772	5.654067	-7.01487	0.000114	0.010546	1.755193
RASL12	-2.35985	4.754222	-7.01423	0.000114	0.010546	1.75457
IFI27	1.869053	7.64254	7.013123	0.000114	0.010546	1.75349
NOX4	-2.20447	6.376168	-7.00451	0.000115	0.010568	1.745069
CCDC88A	1.032941	6.362183	6.98108	0.000117	0.010711	1.72213
ACTN4	-1.32928	8.781708	-6.97814	0.000118	0.010711	1.719244

RBM20	-1.18081	3.469079	-6.96425	0.000119	0.010791	1.705606
ITGB1BP2	-1.87436	3.408296	-6.95679	0.00012	0.010804	1.698267
SLCO2A1	1.967278	2.428404	6.923811	0.000124	0.010818	1.665755
IGFBP3	-1.77561	7.990666	-6.92199	0.000125	0.010818	1.663952
BEND7	-1.4492	3.0175	-6.91987	0.000125	0.010818	1.661865
HIP1R	-1.01921	3.749698	-6.91384	0.000126	0.010818	1.655896
CDH2	-1.73868	4.64642	-6.90716	0.000126	0.010818	1.649288
ZBTB47	-1.08683	4.921516	-6.89217	0.000128	0.010818	1.634422
PDPN	2.109325	4.81493	6.887903	0.000129	0.010818	1.630192
MRAP2	-2.76543	5.234057	-6.87975	0.00013	0.010818	1.622096
FAM19A5	-2.68613	2.167173	-6.87974	0.00013	0.010818	1.622086
GDF7	-1.68611	4.764697	-6.87614	0.00013	0.010818	1.618505
HIST1H2BF	1.351773	3.626597	6.875233	0.000131	0.010818	1.617604
FABP4	3.549138	3.686191	6.868744	0.000131	0.010818	1.611149
NEXN	-2.17857	5.529595	-6.8622	0.000132	0.010818	1.604639
PXDC1	-1.442	6.392645	-6.86197	0.000132	0.010818	1.6044
FBLIM1	-1.40637	8.429979	-6.85998	0.000133	0.010818	1.602426
INPP5A	-1.33599	4.9749	-6.83722	0.000136	0.010957	1.579708
PLXNB1	-1.46172	4.726233	-6.83612	0.000136	0.010957	1.578608
ARHGEF37	-1.27636	2.988415	-6.82295	0.000138	0.011031	1.565435
MKI67	1.778361	1.908994	6.815594	0.000139	0.011031	1.55807
RGS4	-1.21941	6.613954	-6.80494	0.00014	0.011058	1.54739
GALNT2	-1.03105	6.146758	-6.78711	0.000143	0.01115	1.529472
SNX10	2.120006	4.562994	6.780614	0.000144	0.01115	1.522936
PLA2G7	2.140446	3.594932	6.76746	0.000146	0.01115	1.509681
GYPE	-1.15737	1.908525	-6.75511	0.000147	0.01115	1.49722
GNB3	-1.23754	2.179005	-6.75388	0.000148	0.01115	1.495974
AQP1	-1.23069	9.331342	-6.75333	0.000148	0.01115	1.495421
PRDM16	-2.01583	4.089707	-6.73794	0.00015	0.01115	1.47985
ALDH1B1	-2.16043	6.304679	-6.73484	0.000151	0.01115	1.476715
ARHGEF3	1.331303	4.003604	6.733306	0.000151	0.01115	1.475162
DPP4	2.01383	3.417129	6.722545	0.000152	0.01115	1.464254
OSR1	-2.41263	4.453512	-6.72234	0.000152	0.01115	1.464042
ANTXR1	-1.05687	8.310956	-6.71439	0.000154	0.01115	1.455977
EPS8L2	-1.23595	4.567686	-6.71386	0.000154	0.01115	1.455436
ATRNL1	-3.83769	4.81271	-6.70879	0.000155	0.011151	1.450289
CAP2	-1.73008	4.593152	-6.69291	0.000157	0.011166	1.43413
PTHLH	-1.49163	3.934162	-6.68426	0.000159	0.011181	1.425315
COL18A1	-1.76723	7.096274	-6.67527	0.00016	0.011181	1.416153
CXADR	1.878078	1.926885	6.666363	0.000161	0.011181	1.407053
ERBB2	-1.31859	5.046951	-6.66266	0.000162	0.011181	1.403269
LBR	1.224941	5.119589	6.661069	0.000162	0.011181	1.401641
DOK7	-2.3309	1.744725	-6.65796	0.000163	0.011181	1.398463
IGFBP7	-1.22395	11.91498	-6.65401	0.000164	0.011181	1.394424
NRK	-2.02294	2.445827	-6.64961	0.000164	0.011181	1.389911
SDCBP	1.111554	7.837397	6.646629	0.000165	0.011181	1.386861
CEP55	1.309572	1.940434	6.629682	0.000168	0.011204	1.369479
SLC6A6	1.532907	5.502461	6.625605	0.000168	0.011204	1.365291
PDLIM7	-1.4198	7.770921	-6.62429	0.000169	0.011204	1.363943
CRIM1	-1.68663	8.117778	-6.60634	0.000172	0.011362	1.345478
GJA1	-1.36216	7.907256	-6.59301	0.000174	0.011414	1.33173

GPR176	-1.0286	5.717912	-6.57614	0.000177	0.011563	1.314308
GRK3	1.162976	4.093203	6.567106	0.000179	0.011588	1.304962
COL4A5	-1.96397	4.609184	-6.55781	0.000181	0.011588	1.295336
RGCC	1.742469	6.268119	6.549486	0.000182	0.011588	1.2867
LIMS2	-1.41548	5.380028	-6.54645	0.000183	0.011588	1.283553
PACSIN3	-2.09375	3.048689	-6.54515	0.000183	0.011588	1.2822
MREG	1.426264	3.114666	6.538906	0.000184	0.011588	1.275715
GRIK5	-1.43776	2.642294	-6.53386	0.000185	0.011588	1.270466
IGSF9B	-2.17029	2.910617	-6.52894	0.000186	0.011588	1.265349
LGALS9	1.852393	6.07873	6.522493	0.000188	0.011588	1.258642
TMEM206	1.323039	3.835136	6.522106	0.000188	0.011588	1.258239
MCAM	-2.55222	7.745801	-6.51538	0.000189	0.011588	1.251237
INMT-FAM1	-1.44171	3.301334	-6.51349	0.000189	0.011588	1.249265
PDGFA	-1.07143	7.076117	-6.50781	0.00019	0.011588	1.243338
ARHGEF26	-1.82242	4.205974	-6.5069	0.000191	0.011588	1.242392
EXT1	-1.58816	6.748618	-6.50474	0.000191	0.011588	1.240135
APLNR	3.002664	2.973419	6.495921	0.000193	0.011588	1.230925
IFNGR1	1.074929	7.605469	6.493596	0.000193	0.011588	1.228495
ZNF385A	1.45652	4.706349	6.48361	0.000195	0.011588	1.21805
S100A10	1.475298	7.7544	6.48106	0.000196	0.011588	1.215381
FSCN2	-1.17549	1.648685	-6.47737	0.000197	0.011588	1.211522
CDH13	-1.20769	7.860862	-6.47086	0.000198	0.011588	1.204695
ADCY3	1.148446	5.23364	6.462324	0.0002	0.011645	1.195741
ARVCF	-1.745	5.436425	-6.44898	0.000203	0.011762	1.181723
IL5	-1.45374	1.355392	-6.44062	0.000204	0.011816	1.17293
BID	1.769819	4.991457	6.429398	0.000207	0.011816	1.16111
DMTN	-1.80825	3.659008	-6.42852	0.000207	0.011816	1.160184
PLEKHG3	-1.08727	4.606877	-6.41928	0.000209	0.011816	1.150433
CKS2	1.283795	4.281736	6.417474	0.000209	0.011816	1.148531
BMP6	-2.02627	3.211734	-6.41643	0.00021	0.011816	1.147431
ARHGEF9	-1.36215	5.220666	-6.41438	0.00021	0.011816	1.145268
TEAD3	-1.41489	3.597197	-6.40401	0.000212	0.011858	1.1343
PARM1	-2.07194	4.910217	-6.40363	0.000213	0.011858	1.133904
REEP2	-1.15939	3.052233	-6.39599	0.000214	0.011909	1.125815
C1orf198	-1.04468	7.255911	-6.38282	0.000217	0.011983	1.111868
CSTB	1.857702	5.935366	6.373941	0.000219	0.012006	1.102441
PRELP	-1.10547	6.486531	-6.3738	0.000219	0.012006	1.102295
SLC39A8	1.980981	4.655813	6.364881	0.000221	0.012046	1.092816
PLD3	1.107291	8.529129	6.363003	0.000222	0.012046	1.09082
PPP1R13L	-1.1917	4.561893	-6.35671	0.000223	0.012046	1.084128
TNFSF10	1.706935	5.248949	6.356417	0.000223	0.012046	1.083814
NINL	-1.07358	2.97744	-6.34285	0.000227	0.012175	1.069367
TUB	-1.2267	2.839645	-6.33734	0.000228	0.012202	1.063488
FAM149A	-1.17578	3.582167	-6.32301	0.000232	0.012211	1.048185
SMOC2	-1.76	7.877646	-6.32118	0.000232	0.012211	1.046232
HIST1H2AI	1.796877	6.410583	6.317433	0.000233	0.012211	1.042221
CIDEB	1.35695	3.774511	6.313898	0.000234	0.012211	1.038439
ADRA2A	1.146829	1.869848	6.312997	0.000234	0.012211	1.037475
CCR1	2.277089	5.076397	6.307182	0.000236	0.012211	1.031249
FABP5	3.068711	6.423828	6.307096	0.000236	0.012211	1.031158
CCDC8	-1.55493	3.806797	-6.30267	0.000237	0.012211	1.02642

HABP4	-1.01615	5.268856	-6.30053	0.000237	0.012211	1.024126
C11orf52	-1.34003	1.629323	-6.29647	0.000238	0.012211	1.019765
CYP1B1	2.109337	4.218579	6.295534	0.000239	0.012211	1.018764
RHOD	-1.10971	4.541289	-6.29227	0.000239	0.012211	1.015263
WBSCR17	-2.76505	2.135786	-6.28277	0.000242	0.012285	1.005061
KRT7	-2.72072	4.332348	-6.28003	0.000243	0.012285	1.002112
MATK	2.264331	3.458115	6.269236	0.000245	0.012337	0.990506
NCEH1	1.165796	4.398619	6.266227	0.000246	0.012337	0.987266
PRSS35	-2.21897	3.147566	-6.26104	0.000248	0.012363	0.981679
DLGAP1	-3.08631	2.466779	-6.2546	0.000249	0.012393	0.974737
HIST1H2BM	1.957366	3.402023	6.246963	0.000251	0.012393	0.966496
RGN	-1.1707	4.118327	-6.2463	0.000252	0.012393	0.965776
HSPB1	-1.27679	9.043734	-6.24594	0.000252	0.012393	0.96539
ELN	-3.25315	8.593469	-6.24189	0.000253	0.012405	0.961015
INMT	-1.21144	6.905241	-6.2319	0.000255	0.012496	0.950223
RAB23	-1.57566	5.398725	-6.22714	0.000257	0.012518	0.945065
GEM	-2.0433	7.835651	-6.22278	0.000258	0.012528	0.940352
PAM	-1.33782	9.250003	-6.21696	0.00026	0.012528	0.934047
STEAP4	3.320272	5.891938	6.216748	0.00026	0.012528	0.933813
PEBP4	-1.61105	2.012843	-6.21299	0.000261	0.012528	0.929745
CTSL	2.243141	8.664889	6.20537	0.000263	0.012528	0.921472
RFTN1	-1.09056	6.360686	-6.20485	0.000263	0.012528	0.920911
IL1RN	1.965889	3.621406	6.196973	0.000265	0.01259	0.912353
TFEC	1.840454	5.040886	6.190068	0.000267	0.012607	0.904846
NCAPH	1.459036	1.784167	6.188191	0.000268	0.012607	0.902805
LPL	2.143046	3.972472	6.181503	0.00027	0.012607	0.895527
MYLIP	-1.80882	6.423719	-6.17587	0.000271	0.012607	0.88939
CXorf21	1.34221	4.182784	6.174798	0.000272	0.012607	0.888223
MMP1	4.444223	2.98722	6.166099	0.000274	0.012607	0.878737
SYNPO	-1.594	6.491873	-6.16424	0.000275	0.012607	0.876706
NPR3	-3.03472	4.110748	-6.15964	0.000276	0.012607	0.871685
PTRF	-1.04355	8.576834	-6.15752	0.000277	0.012607	0.869375
HIST1H2AJ	1.95731	6.011926	6.156484	0.000277	0.012607	0.86824
SNX25	-1.35728	5.078263	-6.15404	0.000278	0.012607	0.865572
FGF2	-1.41843	4.112527	-6.1539	0.000278	0.012607	0.86542
MRVI1	-1.71475	6.824957	-6.14294	0.000281	0.012607	0.853431
TCEAL4	-1.15491	6.970057	-6.1418	0.000282	0.012607	0.852188
FSTL3	-1.9434	5.634745	-6.14094	0.000282	0.012607	0.851238
CNN1	-3.33351	6.898504	-6.1393	0.000283	0.012607	0.849448
RPRML	-2.2581	2.163873	-6.13667	0.000283	0.012607	0.846565
GRIA3	-1.69195	2.031153	-6.10698	0.000293	0.012903	0.813992
NEO1	-1.68963	5.927	-6.0885	0.000299	0.013059	0.793644
CELSR1	1.678233	2.182909	6.084259	0.0003	0.013059	0.788969
ITGA3	-1.10011	6.440913	-6.08209	0.000301	0.013059	0.78658
OXTR	-2.19433	2.808147	-6.07599	0.000303	0.013108	0.779844
TCEA3	-1.33744	5.089682	-6.06594	0.000306	0.013213	0.768746
SECTM1	1.305126	4.18255	6.056168	0.00031	0.013316	0.757935
SCX	-2.85147	3.046596	-6.05007	0.000312	0.013366	0.751185
C3orf52	-1.04385	2.945836	-6.03623	0.000316	0.013491	0.735835
TREX2	-1.38708	2.556245	-6.03193	0.000318	0.013515	0.731067
WTIP	-1.76579	5.789672	-6.02807	0.000319	0.013528	0.726773

LDB3	-2.71867	3.389025	-6.02566	0.00032	0.013528	0.724099
GPR17	-1.48136	3.059917	-6.01652	0.000323	0.013626	0.713934
FAM46B	-1.92856	3.521377	-6.01182	0.000325	0.01365	0.70871
TMEM130	-1.66499	5.504135	-5.99939	0.00033	0.01365	0.694855
PDZRN3	-2.48733	4.448984	-5.99837	0.00033	0.01365	0.693726
FTL	1.619389	12.7433	5.995351	0.000331	0.01365	0.690354
MGP	-1.47744	13.33589	-5.99497	0.000331	0.01365	0.689934
DMXL2	1.246128	5.991536	5.993918	0.000332	0.01365	0.688755
MAGED2	-1.0405	7.352313	-5.98606	0.000334	0.013667	0.679986
RASGEF1B	1.20785	5.669361	5.984554	0.000335	0.013667	0.6783
CXCL5	1.996733	1.61863	5.98364	0.000335	0.013667	0.677279
DYSF	1.743272	3.074809	5.98247	0.000336	0.013667	0.675972
TCTEX1D1	1.714921	2.297312	5.970655	0.00034	0.013734	0.662759
NOV	-2.33289	9.026344	-5.97042	0.00034	0.013734	0.6625
CD36	4.659112	6.440157	5.955614	0.000346	0.013923	0.645908
TLR6	1.183751	3.598625	5.950486	0.000348	0.013925	0.640155
SLC16A6	1.310003	2.400054	5.947617	0.000349	0.01393	0.636935
HIST1H3G	1.568204	2.95457	5.942986	0.000351	0.013959	0.631735
DACT3	-1.80869	4.930725	-5.93594	0.000354	0.013963	0.623813
FAM110C	-1.03488	1.688361	-5.93157	0.000355	0.013963	0.618906
MANSC1	-1.47958	3.857796	-5.92993	0.000356	0.013963	0.617057
HSPB8	-2.16336	5.572971	-5.92764	0.000357	0.013963	0.614476
TRABD2B	-1.70754	2.058056	-5.92577	0.000358	0.013963	0.612372
CTPS1	-1.20428	5.257049	-5.91998	0.00036	0.013977	0.605854
SLC31A2	1.307579	5.119024	5.916591	0.000361	0.013987	0.602035
HIF3A	-1.97128	2.519784	-5.91134	0.000364	0.013987	0.596113
CYFIP2	-1.53448	5.846502	-5.90928	0.000364	0.013987	0.593793
LTBP1	-1.60528	9.350051	-5.90837	0.000365	0.013987	0.592759
LMOD1	-2.47988	6.992737	-5.90727	0.000365	0.013987	0.591518
TGFB1I1	-1.27337	5.631857	-5.90492	0.000366	0.013987	0.588871
LRRC8A	-1.01201	5.002447	-5.89727	0.000369	0.014053	0.580232
VAV3	1.864454	3.68157	5.895988	0.00037	0.014053	0.578782
PLXNB3	-1.15405	2.115569	-5.88516	0.000374	0.014187	0.56654
KYNU	2.052797	4.682943	5.875461	0.000379	0.01425	0.555552
CST6	-1.61387	5.125788	-5.87476	0.000379	0.01425	0.554753
MYH11	-2.63613	8.901054	-5.87422	0.000379	0.01425	0.554142
IGFBP6	-1.30653	7.934182	-5.86868	0.000381	0.014301	0.547861
MPP1	1.346266	5.967614	5.853787	0.000388	0.014471	0.530957
CHST13	1.234527	1.61307	5.853009	0.000388	0.014471	0.530072
SMAD9	-1.71131	3.835095	-5.85128	0.000389	0.014471	0.528103
ACTA2	-2.48475	11.33313	-5.84625	0.000391	0.014506	0.522383
PRKCDBP	-1.02426	7.730409	-5.83458	0.000396	0.014632	0.509104
CDK1	1.463671	3.136481	5.817256	0.000404	0.014884	0.489344
CA2	2.392471	3.556188	5.812487	0.000406	0.014926	0.483898
MAP3K7CL	-1.47415	4.997887	-5.79692	0.000414	0.015076	0.466097
CLDN23	1.480386	2.542005	5.791059	0.000416	0.015099	0.459382
CAMK2G	-1.17158	5.510387	-5.78313	0.00042	0.015198	0.450297
IL10	1.5307	2.471874	5.768827	0.000427	0.015391	0.433874
TINAGL1	-1.79663	7.60567	-5.7676	0.000428	0.015391	0.432462
PCBP4	-1.01519	5.091441	-5.7622	0.00043	0.015447	0.426251
DSTN	-1.94373	9.940422	-5.75196	0.000435	0.015533	0.414477

ADCY9	-1.0832	4.286371	-5.75135	0.000436	0.015533	0.413771
TPH1	-4.10276	3.869457	-5.75036	0.000436	0.015533	0.412636
ARHGEF17	-1.34058	5.853395	-5.74541	0.000439	0.015551	0.406926
GPR65	1.023536	3.990281	5.733828	0.000445	0.015642	0.393568
OAS3	1.07933	4.428544	5.73125	0.000446	0.015649	0.390593
CMIP	1.300661	4.151317	5.725247	0.000449	0.015679	0.383659
SNTA1	-1.28813	4.965565	-5.72328	0.00045	0.015679	0.381391
CTSK	1.79632	7.997048	5.723191	0.00045	0.015679	0.381282
COL6A3	1.940773	8.180641	5.716166	0.000454	0.015695	0.373159
MAP6	-1.29156	2.906959	-5.71559	0.000454	0.015695	0.37249
LHFPL2	1.617681	6.54801	5.714972	0.000454	0.015695	0.371778
GSG1L	-2.83193	1.955586	-5.71238	0.000456	0.015695	0.368784
TREM1	2.77096	5.92575	5.71169	0.000456	0.015695	0.36798
KANK2	-1.01764	7.195349	-5.70952	0.000457	0.015697	0.36547
WASF3	-1.06076	2.795205	-5.70333	0.00046	0.015771	0.358299
FLNA	-1.44521	10.1588	-5.70052	0.000462	0.015784	0.355036
EFHD1	-2.30098	5.469204	-5.69238	0.000466	0.015894	0.345604
MYOCD	-3.65198	3.433285	-5.67291	0.000477	0.016087	0.322977
SORBS2	-1.9001	7.073984	-5.67178	0.000477	0.016087	0.321664
ADCY5	-2.30298	3.819451	-5.66955	0.000479	0.016087	0.319069
METTL24	-1.69417	3.16584	-5.65729	0.000485	0.016172	0.304789
PDE6G	1.812711	1.602191	5.656264	0.000486	0.016172	0.303591
CTTN	-1.00103	7.407212	-5.65115	0.000489	0.016172	0.29763
SMTN	-1.8839	5.988961	-5.64894	0.00049	0.016172	0.295044
DKK3	-1.25363	8.612416	-5.64371	0.000493	0.01623	0.288944
VCAN	-1.27553	10.54307	-5.64053	0.000495	0.016253	0.285232
PTGER3	-2.0706	4.175775	-5.62141	0.000506	0.016502	0.262868
CAPG	1.762108	8.33081	5.618466	0.000508	0.016502	0.259416
SHTN1	1.014927	4.790123	5.617509	0.000508	0.016502	0.258295
EVI2B	1.231884	4.789305	5.615323	0.00051	0.016506	0.255732
ABI3BP	1.233701	6.194775	5.61342	0.000511	0.016506	0.253501
ANKRD6	-1.81973	5.58173	-5.60621	0.000515	0.016552	0.245041
SCHIP1	-1.47355	4.961464	-5.60517	0.000516	0.016552	0.243829
ANXA3	-1.61276	2.977343	-5.59508	0.000522	0.016672	0.231972
GM2A	1.138888	5.795196	5.587574	0.000526	0.016744	0.22315
MYL9	-1.85001	9.420017	-5.57207	0.000536	0.016798	0.204897
SEL1L3	1.664966	4.45646	5.571518	0.000536	0.016798	0.204246
NR2F1	1.086814	1.321836	5.565146	0.00054	0.016849	0.196732
PLIN2	2.311834	6.156205	5.55972	0.000544	0.01689	0.190329
RRM2	1.570996	2.588543	5.55723	0.000545	0.01689	0.187388
ST6GAL2	-2.92173	5.018948	-5.55241	0.000548	0.01689	0.181701
TIAM1	1.347237	3.855409	5.550487	0.00055	0.01689	0.179422
VCL	-1.25974	7.52535	-5.54811	0.000551	0.01689	0.176612
SMARCA1	-1.07858	5.219576	-5.54431	0.000554	0.01689	0.172119
FAM129A	-1.27752	7.26039	-5.54412	0.000554	0.01689	0.171899
ADAMTS4	1.577738	2.313792	5.542439	0.000555	0.01689	0.169905
RRAD	-1.6824	5.199353	-5.5415	0.000555	0.01689	0.1688
DDX60L	1.933039	5.390891	5.539753	0.000557	0.01689	0.166727
PRKG1	-1.69477	6.564881	-5.53915	0.000557	0.01689	0.166008
ASPM	1.213385	1.671201	5.539138	0.000557	0.01689	0.165999
HAS2	1.138133	1.876612	5.526099	0.000565	0.017005	0.150553

SAMD14	-1.17081	2.398351	-5.52351	0.000567	0.017005	0.14748
RPS6KA6	-1.97158	2.532228	-5.5225	0.000568	0.017005	0.146288
PTGDS	3.972388	5.758282	5.511646	0.000575	0.017187	0.133402
PCDH7	-1.92192	7.092787	-5.50513	0.00058	0.017282	0.125661
GAREM1	-1.591	2.87436	-5.50339	0.000581	0.017282	0.123587
ACADL	-2.03702	3.781762	-5.49749	0.000585	0.017366	0.116567
CD4	1.599894	5.657815	5.488584	0.000591	0.017476	0.105968
LCP1	1.915044	7.869591	5.486675	0.000592	0.01748	0.103692
MMP9	5.017666	6.349952	5.48201	0.000596	0.017539	0.098132
MYOM1	-2.12397	3.197945	-5.46214	0.00061	0.017775	0.074412
SKAP2	1.291445	4.80966	5.46202	0.00061	0.017775	0.074267
HMGA1	1.536804	4.012051	5.457101	0.000613	0.017806	0.068384
PDLIM3	-1.88246	8.351975	-5.45303	0.000616	0.017819	0.063508
KAZALD1	-1.54228	3.851151	-5.44182	0.000624	0.017937	0.050092
TMEM25	-1.19202	4.279802	-5.44041	0.000626	0.017937	0.048398
MAOB	-1.45069	4.715872	-5.44011	0.000626	0.017937	0.048041
NRP2	1.051864	6.523159	5.440093	0.000626	0.017937	0.048018
PTPRZ1	-1.44676	2.802122	-5.43661	0.000628	0.017937	0.043843
CNTN3	-2.26364	2.385256	-5.43568	0.000629	0.017937	0.042727
CLEC5A	2.513759	5.383827	5.43496	0.00063	0.017937	0.041862
CNTN4	-3.13099	3.692676	-5.43385	0.00063	0.017937	0.040537
LIMS4	-1.46004	3.953298	-5.4307	0.000633	0.017944	0.036747
ACP5	2.626977	6.120708	5.430157	0.000633	0.017944	0.036099
ASPN	-1.95917	8.076186	-5.42666	0.000636	0.017949	0.031907
PLCD3	-1.23644	4.664826	-5.4266	0.000636	0.017949	0.031832
SACS	-1.04292	4.850188	-5.42335	0.000638	0.017983	0.027925
GPR183	1.286307	4.709026	5.412206	0.000647	0.018151	0.014529
HIST1H4L	1.264039	6.20619	5.411835	0.000647	0.018151	0.014081
ITGA8	-2.03812	8.683836	-5.40887	0.000649	0.018151	0.01051
DMKN	-1.04061	5.011017	-5.40321	0.000654	0.018202	0.003705
C1orf186	1.007934	3.355133	5.390562	0.000664	0.018357	-0.01155
VWA1	1.491409	3.977461	5.38946	0.000665	0.018357	-0.01288
FGFRL1	-1.37042	4.048963	-5.38839	0.000665	0.018357	-0.01417
KANK1	-1.87389	6.153322	-5.38793	0.000666	0.018357	-0.01472
VASN	-1.30287	5.357947	-5.37488	0.000676	0.018502	-0.03048
FCER1G	1.940429	7.544552	5.370396	0.00068	0.018566	-0.03591
GPLD1	-2.00819	3.078747	-5.36496	0.000684	0.018601	-0.04249
MYO5B	-1.83657	2.382184	-5.35302	0.000694	0.018705	-0.05694
RASL11B	-1.7646	3.183452	-5.33507	0.000709	0.019003	-0.07872
RRAS2	-1.18533	4.961973	-5.31846	0.000723	0.019241	-0.09893
SCUBE3	-2.52754	3.655032	-5.31171	0.000729	0.01929	-0.10715
CD79B	1.641664	2.33624	5.305938	0.000734	0.019388	-0.11419
C9orf3	-1.41514	7.916032	-5.30271	0.000737	0.019428	-0.11812
DIXDC1	-1.01625	5.878292	-5.29915	0.00074	0.019475	-0.12247
DRAM1	1.31596	5.528638	5.282655	0.000755	0.019748	-0.14262
SPECC1	-1.1581	6.688267	-5.28105	0.000757	0.019748	-0.14458
SUGCT	-1.5386	7.056266	-5.27395	0.000763	0.019856	-0.15327
TMEM35A	-1.26629	1.431624	-5.26547	0.000771	0.019938	-0.16367
SCARF1	1.478115	3.760163	5.262921	0.000773	0.019938	-0.16679
RAI2	-1.16353	2.762754	-5.26237	0.000774	0.019938	-0.16747
LTBP4	-1.56242	7.73575	-5.26005	0.000776	0.019938	-0.17031

MFAP4	-1.88666	7.662379	-5.25307	0.000783	0.019988	-0.17888
TC2N	-1.65914	5.642244	-5.24274	0.000792	0.020012	-0.19157
ALPK3	-1.20705	4.451976	-5.23928	0.000796	0.020013	-0.19583
MYO5C	1.008679	1.674276	5.234706	0.0008	0.020043	-0.20145
TLR8	1.313249	3.476926	5.232204	0.000803	0.020044	-0.20453
HIST1H2AH	1.258414	6.36972	5.225719	0.000809	0.020067	-0.21251
GPR68	1.891066	2.960934	5.225516	0.000809	0.020067	-0.21276
MELTF	1.853579	3.055984	5.220419	0.000814	0.020151	-0.21904
IRF6	-1.41393	2.178731	-5.21653	0.000818	0.020163	-0.22384
FHOD3	-1.27147	3.72983	-5.21445	0.00082	0.020163	-0.2264
TTLL7	-1.75732	5.962245	-5.21207	0.000823	0.020187	-0.22934
CFI	1.47925	5.257036	5.206604	0.000828	0.020284	-0.23609
HMMR	1.553086	1.793087	5.205311	0.000829	0.020284	-0.23769
NCKAP1L	1.429632	6.353481	5.20299	0.000832	0.020306	-0.24055
HACD1	-2.25906	5.026006	-5.19985	0.000835	0.020349	-0.24443
HOXD8	1.192588	1.482315	5.194975	0.00084	0.020405	-0.25046
ACP2	1.23253	5.424616	5.188735	0.000846	0.020521	-0.25818
DUSP10	1.238594	3.618143	5.186499	0.000849	0.02054	-0.26095
PPP1R12B	-2.42929	7.819621	-5.18332	0.000852	0.020552	-0.26489
CALML4	1.505281	2.995756	5.176677	0.000859	0.020681	-0.27312
TPP1	1.121293	7.091048	5.172817	0.000863	0.020712	-0.2779
SCD	2.130108	5.219514	5.169088	0.000867	0.020758	-0.28253
LRRC15	2.343355	2.203065	5.165276	0.000871	0.020799	-0.28726
ITGA10	-1.55902	6.116867	-5.163	0.000873	0.020822	-0.29008
DEPTOR	1.439814	4.208417	5.159324	0.000877	0.020881	-0.29465
TIMP3	1.883976	8.059157	5.154042	0.000883	0.020981	-0.30121
PGM5	-1.81712	5.438898	-5.1517	0.000885	0.021007	-0.30412
PRUNE2	-1.7869	6.760181	-5.14169	0.000896	0.02116	-0.31658
SYNM	-1.53865	5.211001	-5.13717	0.000901	0.021173	-0.32222
ITPR1	-1.06535	6.166114	-5.13333	0.000905	0.021187	-0.327
COTL1	1.358829	6.699296	5.132581	0.000906	0.021187	-0.32793
MSRB3	-1.31357	6.875903	-5.1293	0.00091	0.021238	-0.33202
DAPK3	-1.00948	6.312477	-5.12326	0.000917	0.021249	-0.33955
CYB5R4	1.111338	5.351358	5.122425	0.000918	0.021249	-0.3406
RAC2	1.787585	5.08885	5.112634	0.000929	0.021249	-0.35282
KDR	2.107498	3.270874	5.111507	0.00093	0.021249	-0.35423
FBXL22	-1.79762	2.994515	-5.10995	0.000932	0.021249	-0.35617
CD84	1.59934	5.797478	5.106498	0.000936	0.021249	-0.36049
SPP1	3.642205	11.86263	5.101833	0.000941	0.021306	-0.36633
KIF11	1.106008	1.923704	5.095905	0.000948	0.021343	-0.37375
SLC43A2	1.156411	4.862388	5.094024	0.00095	0.021343	-0.37611
CLEC7A	1.413381	6.567051	5.092274	0.000952	0.021343	-0.3783
CPVL	1.858595	6.451496	5.091476	0.000953	0.021343	-0.3793
QRFP	-1.11569	1.800216	-5.09146	0.000953	0.021343	-0.37932
CRIP2	-1.3038	8.472187	-5.08966	0.000955	0.021355	-0.38157
RBPMS	-1.3983	6.090312	-5.08788	0.000957	0.021355	-0.3838
TBX18	-1.20761	5.246526	-5.08717	0.000958	0.021355	-0.38469
CREG1	1.105768	7.782299	5.080573	0.000966	0.021478	-0.39297
FAM49A	1.116189	3.71235	5.075969	0.000972	0.021478	-0.39874
RGS5	-2.98211	9.741741	-5.07257	0.000976	0.021478	-0.40301
OLFML2A	2.53536	2.705579	5.07086	0.000978	0.021478	-0.40516

TYROBP	1.824721	8.730084	5.06917	0.00098	0.021478	-0.40728
GALNT6	1.800676	3.964343	5.066958	0.000983	0.021478	-0.41006
LLNLF-187	-1.55286	1.659494	-5.06602	0.000984	0.021478	-0.41124
CHSY3	-1.31157	4.898659	-5.06258	0.000988	0.021478	-0.41556
SBSPON	-2.60004	2.770397	-5.06082	0.00099	0.021478	-0.41778
TRAT1	1.090632	2.339046	5.059661	0.000991	0.021478	-0.41924
CALD1	-1.55713	9.556116	-5.05834	0.000993	0.021478	-0.4209
COL16A1	-1.13793	7.001916	-5.05762	0.000994	0.021478	-0.4218
NT5DC3	-1.64995	3.968377	-5.05404	0.000998	0.021478	-0.42631
NABP1	1.64153	6.812282	5.053429	0.000999	0.021478	-0.42708
CHRDL1	-1.22644	2.136044	-5.05033	0.001003	0.021478	-0.43098
ADRA1A	-1.30022	2.201124	-5.04764	0.001006	0.021488	-0.43437
PECAM1	1.572789	7.266699	5.046019	0.001008	0.021488	-0.43641
HS2ST1	1.00941	4.082927	5.035727	0.001021	0.021643	-0.44939
NOD2	1.125767	3.26851	5.032111	0.001026	0.021676	-0.45395
FOXC2	-1.41877	2.786234	-5.02913	0.00103	0.021692	-0.45771
NHSL1	1.033763	2.318649	5.027605	0.001032	0.021701	-0.45964
GRIA1	-2.15691	4.128661	-5.025	0.001035	0.021739	-0.46293
HIST1H2BE	1.342655	5.04727	5.016652	0.001046	0.021895	-0.47348
KLC2	-1.0175	3.928111	-5.01619	0.001046	0.021895	-0.47406
PARPBP	1.112012	2.086561	5.014514	0.001048	0.021896	-0.47619
EDN1	-1.0231	3.629541	-5.01119	0.001053	0.021913	-0.48039
TNS1	-1.29376	8.463829	-5.00894	0.001056	0.02192	-0.48325
CYTIP	1.497828	5.136712	5.002761	0.001064	0.02193	-0.49106
ARAP2	1.174764	4.340297	5.001005	0.001066	0.02193	-0.49329
TMEM8B	-1.19252	2.884054	-5.00044	0.001067	0.02193	-0.49401
HSPA12B	1.54886	1.848713	4.998813	0.001069	0.021933	-0.49607
ME2	1.159783	6.002368	4.998019	0.00107	0.021933	-0.49707
PTAFR	1.516263	4.504058	4.991055	0.00108	0.02206	-0.50591
ZNF704	-1.55251	5.923014	-4.98244	0.001091	0.022254	-0.51684
CXorf36	1.546601	2.275171	4.978889	0.001096	0.022275	-0.52135
TGFBI	1.326011	8.149988	4.978723	0.001096	0.022275	-0.52156
CAV2	-1.1985	7.018737	-4.9772	0.001098	0.022285	-0.5235
YAP1	-1.33572	6.730365	-4.97573	0.0011	0.022295	-0.52536
SOST	-3.25769	3.993279	-4.97127	0.001107	0.022338	-0.53103
FHL1	-1.94365	9.36062	-4.97078	0.001107	0.022338	-0.53166
ZNF853	-1.2291	2.12951	-4.97076	0.001107	0.022338	-0.53168
CD80	1.572672	4.042167	4.961913	0.00112	0.022355	-0.54294
MAP1B	-1.56938	7.335317	-4.95708	0.001126	0.022355	-0.5491
SLC6A12	1.154443	2.491875	4.956987	0.001126	0.022355	-0.54922
MCTP1	1.702459	5.597614	4.956294	0.001127	0.022355	-0.5501
LRMP	1.187357	4.344151	4.95613	0.001128	0.022355	-0.55031
MARK1	-1.58612	2.495303	-4.95494	0.001129	0.022355	-0.55182
SPEG	-1.9609	5.954799	-4.95374	0.001131	0.022355	-0.55335
TALDO1	1.016551	7.100193	4.953	0.001132	0.022355	-0.5543
WFS1	-1.36802	4.826843	-4.9512	0.001135	0.022355	-0.55659
PPP1R9A	-1.93884	1.737453	-4.94889	0.001138	0.022358	-0.55954
KIF7	-1.04151	3.315781	-4.94236	0.001147	0.022474	-0.56787
TCEAL1	-1.09326	6.012532	-4.94067	0.00115	0.022474	-0.57002
BLNK	1.129593	4.224668	4.937529	0.001154	0.022474	-0.57404
HSPA2	-1.20654	4.333941	-4.91802	0.001183	0.022876	-0.59899

HHEX	1.193504	4.015424	4.914984	0.001187	0.022876	-0.60287
GPRIN3	1.563641	4.293319	4.914879	0.001188	0.022876	-0.603
ADAM33	-1.9645	3.513812	-4.91435	0.001188	0.022876	-0.60368
SLC6A16	-1.09376	1.678258	-4.91212	0.001192	0.022897	-0.60653
NR5A2	1.213236	1.360422	4.910729	0.001194	0.022897	-0.60832
TSPAN11	2.252138	2.442685	4.910415	0.001194	0.022897	-0.60872
TCN2	1.341691	4.906993	4.907406	0.001199	0.022951	-0.61258
IGFBP2	-1.02309	9.816363	-4.89895	0.001212	0.023075	-0.62343
PLCB1	1.742972	3.32575	4.894898	0.001218	0.0231	-0.62862
MYOZ3	-1.05098	1.990092	-4.88975	0.001226	0.023141	-0.63523
ATP6V1A	1.063685	6.459351	4.889317	0.001226	0.023141	-0.63579
HSPB2-C11	-1.16546	3.979436	-4.88826	0.001228	0.023141	-0.63714
TMEM2	1.731305	5.69463	4.884799	0.001233	0.023181	-0.64159
MMP15	1.134901	1.411	4.883337	0.001236	0.023193	-0.64347
BCAT1	2.100215	6.499345	4.881801	0.001238	0.023206	-0.64545
ADGRB2	-1.01414	1.22754	-4.8808	0.00124	0.023206	-0.64673
AMIGO1	-1.03634	2.86373	-4.87075	0.001255	0.02332	-0.65967
PLEK	1.79007	6.020631	4.864097	0.001266	0.023425	-0.66824
APOBR	1.55238	2.375594	4.85839	0.001275	0.023505	-0.6756
NLN	1.031693	4.378416	4.852937	0.001284	0.023575	-0.68263
HIST1H2BC	1.576407	5.162242	4.850298	0.001288	0.023593	-0.68603
CAB39L	-1.26699	4.189246	-4.84374	0.001299	0.023674	-0.6945
LTBP3	-1.04158	7.13335	-4.84364	0.001299	0.023674	-0.69463
SPNS2	1.439351	2.746493	4.843521	0.001299	0.023674	-0.69479
GBP5	1.235631	4.404812	4.840929	0.001304	0.023674	-0.69813
LILRB3	1.725541	4.695571	4.840201	0.001305	0.023674	-0.69908
SV2A	-1.34859	3.139395	-4.83954	0.001306	0.023674	-0.69992
CCDC170	1.031046	2.829626	4.830275	0.001321	0.023924	-0.71191
C1QTNF2	-1.14293	3.625295	-4.82687	0.001327	0.023978	-0.71631
RIMS1	-2.24531	2.491692	-4.82419	0.001332	0.023978	-0.71979
OAS1	1.262708	5.118883	4.821198	0.001337	0.024018	-0.72366
C8orf88	-1.57983	4.322581	-4.81887	0.001341	0.024059	-0.72668
BUB1	1.141626	1.963211	4.815873	0.001346	0.024077	-0.73056
CYYR1	1.775413	2.307679	4.81513	0.001347	0.024077	-0.73153
ACP6	-1.00089	3.748592	-4.81232	0.001352	0.024077	-0.73517
GLUL	1.497599	9.630954	4.81213	0.001352	0.024077	-0.73542
RAMP2	2.444465	3.053655	4.811172	0.001354	0.024077	-0.73666
CACNA2D4	1.790793	4.632812	4.803959	0.001366	0.024216	-0.74602
CSNK2A3	-1.18387	1.38032	-4.79087	0.001389	0.024547	-0.76303
LAPTM5	2.083378	8.265472	4.787914	0.001395	0.024547	-0.76687
PNMA6A	-1.19773	1.672054	-4.78784	0.001395	0.024547	-0.76696
LRRC8D	1.431985	4.065188	4.785518	0.001399	0.024547	-0.76998
OPN3	1.352108	4.082195	4.783929	0.001402	0.024547	-0.77205
LRP2BP	-1.22436	3.218627	-4.78361	0.001402	0.024547	-0.77247
SGCE	-1.29394	5.608506	-4.78231	0.001405	0.024558	-0.77415
HK2	1.40055	3.931	4.779338	0.00141	0.024621	-0.77803
FLNC	-1.91422	3.510495	-4.7743	0.001419	0.024693	-0.78458
CFAP221	-1.56535	1.305889	-4.77332	0.001421	0.024693	-0.78587
ZC2HC1C	-1.18549	1.504525	-4.77248	0.001422	0.024693	-0.78696
MTMR11	-1.27408	4.637092	-4.77172	0.001424	0.024693	-0.78795
NRIP3	1.588022	3.45943	4.768133	0.00143	0.024693	-0.79262

RAB9B	-1.3914	2.35101	-4.76775	0.001431	0.024693	-0.79313
SYDE2	-1.43541	3.013351	-4.76738	0.001432	0.024693	-0.7936
TNFRSF10A	1.187662	3.132333	4.766454	0.001433	0.024693	-0.79481
CFL2	-1.52753	6.027042	-4.76567	0.001435	0.024693	-0.79584
LMCD1	-1.66259	7.852637	-4.76337	0.001439	0.024736	-0.79884
ITGAM	2.076869	5.87312	4.762371	0.001441	0.024738	-0.80014
ATP6AP1L	-1.29658	3.7425	-4.75993	0.001445	0.024785	-0.80332
OAF	1.556281	3.35985	4.758523	0.001448	0.0248	-0.80516
CTGF	-1.06339	9.421354	-4.75654	0.001452	0.024803	-0.80775
C5orf46	-2.23192	5.741257	-4.75079	0.001462	0.024875	-0.81526
CTSB	2.079274	11.18496	4.750738	0.001462	0.024875	-0.81533
TBX2	-1.16951	4.686885	-4.74663	0.00147	0.024941	-0.8207
HDAC11	-1.07782	4.368972	-4.74523	0.001473	0.024956	-0.82252
SOX18	1.419209	1.936176	4.741229	0.00148	0.025055	-0.82776
FAM110A	1.028691	2.645711	4.736891	0.001489	0.02516	-0.83343
TNFSF8	1.666678	3.867163	4.728406	0.001505	0.025291	-0.84454
STC1	1.354851	1.289353	4.726272	0.001509	0.02533	-0.84734
COL4A3	-1.43074	3.627002	-4.72117	0.001519	0.025467	-0.85402
FGF13	-1.12704	3.558851	-4.71643	0.001528	0.025563	-0.86025
MAPK13	2.155847	3.431	4.706706	0.001548	0.025598	-0.87301
SLC22A3	-2.09521	4.951496	-4.70607	0.001549	0.025598	-0.87384
KMO	1.527377	2.878357	4.705241	0.00155	0.025598	-0.87493
SULT1A1	1.225459	3.642282	4.704686	0.001552	0.025598	-0.87566
MEIKIN	1.627203	3.348219	4.704412	0.001552	0.025598	-0.87602
MRGPRF	-1.44766	4.57416	-4.70355	0.001554	0.025598	-0.87715
GRN	1.581537	8.25629	4.702265	0.001556	0.025598	-0.87884
NEURL1B	1.640709	2.494335	4.700557	0.00156	0.025598	-0.88108
KRT17	-1.83987	5.730392	-4.6995	0.001562	0.025598	-0.88248
HS3ST1	1.11796	2.829641	4.697611	0.001566	0.025598	-0.88496
TOP2A	1.424126	3.055496	4.696729	0.001568	0.025598	-0.88612
GPRASP1	-1.27051	4.09865	-4.69654	0.001568	0.025598	-0.88637
TSPAN33	1.149856	5.006725	4.69459	0.001572	0.025598	-0.88893
TPST2	1.215147	5.387579	4.693822	0.001573	0.025598	-0.88994
SLAMF8	2.029242	5.339959	4.689294	0.001583	0.025685	-0.8959
NRP1	1.497246	7.629826	4.688784	0.001584	0.025685	-0.89657
PTK2B	1.107729	5.311757	4.688553	0.001584	0.025685	-0.89687
NCAPG	1.349061	1.893442	4.687546	0.001586	0.025689	-0.8982
SSTR5	-1.76782	1.371138	-4.68424	0.001593	0.025729	-0.90256
LPAR6	1.118794	6.945999	4.683727	0.001594	0.025729	-0.90323
IGF1R	-1.09082	5.382307	-4.67914	0.001604	0.025794	-0.90927
SLC16A10	2.486482	4.471937	4.677435	0.001607	0.025822	-0.91152
ADAMTS14	1.192873	1.413533	4.673909	0.001614	0.025911	-0.91616
LSP1	1.732035	7.250299	4.671572	0.001619	0.02596	-0.91925
DIP2C	-1.04194	4.220125	-4.66931	0.001624	0.025967	-0.92223
SERPINF1	2.145723	6.933399	4.668829	0.001625	0.025967	-0.92286
APOLD1	1.010307	2.413452	4.668772	0.001625	0.025967	-0.92294
COL8A2	-1.06654	5.38812	-4.66528	0.001633	0.026026	-0.92754
RIMKLB	-1.2528	6.215739	-4.66428	0.001635	0.02603	-0.92887
SLC37A2	1.304446	5.567603	4.660562	0.001643	0.026127	-0.93378
FOXS1	-1.57193	3.871504	-4.65682	0.001651	0.026155	-0.93872
CSRP1	-1.5632	8.891572	-4.65629	0.001652	0.026155	-0.93942

CCL21	2.954967	2.717641	4.653255	0.001658	0.0262	-0.94343
KCNMB1	-1.22328	4.392157	-4.64534	0.001675	0.026412	-0.9539
LPP	-1.3571	7.847235	-4.64444	0.001677	0.026412	-0.95509
THSD7A	1.659159	1.907368	4.642854	0.001681	0.026412	-0.95719
CCR5	1.116506	3.404004	4.642345	0.001682	0.026412	-0.95786
GNA15	1.524121	4.157415	4.641742	0.001683	0.026412	-0.95866
COL9A2	-1.76907	3.289651	-4.64136	0.001684	0.026412	-0.95917
ADAM12	1.390768	2.47446	4.639532	0.001688	0.026412	-0.96158
UNC13C	-1.92871	1.781	-4.6367	0.001694	0.026412	-0.96534
CTD-2192J	1.300404	1.58314	4.635172	0.001697	0.026412	-0.96736
C5AR1	1.953258	5.547106	4.635092	0.001698	0.026412	-0.96746
HLA-B	1.139085	10.49504	4.634349	0.001699	0.026412	-0.96845
PNMAL1	-1.71623	3.342544	-4.63245	0.001703	0.026423	-0.97096
ITGA1	-1.60856	7.22919	-4.63128	0.001706	0.026423	-0.97251
TMEM51	1.256146	3.982073	4.627496	0.001714	0.026446	-0.97753
ANPEP	2.688052	4.874097	4.625362	0.001719	0.026482	-0.98036
IQGAP3	1.522615	1.929551	4.622767	0.001725	0.026519	-0.9838
ALS2CL	-1.10079	3.452802	-4.62128	0.001728	0.026519	-0.98577
HSPB7	-2.76532	4.938171	-4.61841	0.001735	0.026531	-0.98958
ADCY6	-1.12299	5.146179	-4.6125	0.001748	0.026708	-0.99743
TSPYL2	-1.0099	4.867612	-4.60911	0.001756	0.026768	-1.00193
LRRC75B	-1.11143	2.657083	-4.60789	0.001759	0.026782	-1.00355
CD68	2.039901	7.855269	4.605925	0.001763	0.02681	-1.00617
HOXA3	-1.24967	2.818999	-4.60545	0.001764	0.02681	-1.0068
CMTM7	1.302688	3.743109	4.601414	0.001774	0.026887	-1.01216
ZNF267	1.12532	5.215346	4.599429	0.001778	0.026887	-1.0148
FILIP1	-1.31443	4.066265	-4.59917	0.001779	0.026887	-1.01515
SLC4A3	-1.00422	1.485625	-4.598	0.001782	0.026887	-1.01671
PLEKHH2	-1.13475	3.96821	-4.5976	0.001782	0.026887	-1.01724
HK3	2.244827	4.438634	4.595331	0.001788	0.026887	-1.02026
AL513122.	2.262348	2.455471	4.594907	0.001789	0.026887	-1.02082
SVIL	-1.55195	7.162935	-4.59057	0.001799	0.026887	-1.0266
GRAP	1.425972	2.630289	4.589154	0.001802	0.026887	-1.02848
ST8SIA4	1.318366	5.35393	4.588796	0.001803	0.026887	-1.02896
PKD2	-1.35086	6.994956	-4.58789	0.001805	0.026887	-1.03017
PATJ	-1.26991	4.466126	-4.5876	0.001806	0.026887	-1.03055
PPM1N	1.185272	1.908712	4.585576	0.001811	0.026887	-1.03324
BDNF	-1.94262	1.588464	-4.5852	0.001812	0.026887	-1.03375
IGSF21	1.295558	4.38435	4.584756	0.001813	0.026887	-1.03434
MX2	1.435238	4.487425	4.584721	0.001813	0.026887	-1.03438
CEMIP	2.608162	5.940318	4.578468	0.001828	0.026968	-1.04272
CCL8	2.025369	4.754661	4.578416	0.001828	0.026968	-1.04279
MTURN	-1.01314	5.391818	-4.57403	0.001838	0.027006	-1.04864
FAM105A	1.069208	4.607735	4.572797	0.001841	0.027006	-1.05029
PIK3AP1	1.561927	5.861806	4.572636	0.001841	0.027006	-1.0505
CARD10	1.016148	1.749798	4.566784	0.001856	0.027158	-1.05831
UHRF1	1.027975	2.37602	4.565929	0.001858	0.02716	-1.05945
FAM47E-ST	-1.73644	2.598415	-4.56332	0.001864	0.027225	-1.06293
PGR	-1.45745	3.721347	-4.55613	0.001882	0.027423	-1.07254
AC124312.	-1.35828	2.551009	-4.55314	0.001889	0.027423	-1.07654
NLRP3	1.289029	3.973708	4.552697	0.00189	0.027423	-1.07714

CLIC2	1.144691	4.778962	4.545371	0.001908	0.027479	-1.08694
SRPK3	-2.2712	1.862426	-4.54421	0.001911	0.027479	-1.08849
NPC2	1.144571	9.354116	4.544024	0.001912	0.027479	-1.08874
COLQ	-1.24064	3.001772	-4.54245	0.001916	0.027479	-1.09085
P2RX7	1.295013	4.546392	4.542182	0.001916	0.027479	-1.09121
ARL15	1.004609	5.112546	4.541596	0.001918	0.027479	-1.09199
OASL	1.08262	2.456809	4.541538	0.001918	0.027479	-1.09207
SORBS1	-1.70922	7.015445	-4.53884	0.001925	0.027549	-1.09569
LMO7	-1.23745	7.441402	-4.53641	0.001931	0.027582	-1.09894
FXYD6	1.752613	3.752685	4.534944	0.001935	0.027607	-1.10091
FERMT2	-1.24329	6.833407	-4.53273	0.00194	0.027636	-1.10388
TMEM56	-2.50843	3.151111	-4.53066	0.001946	0.02768	-1.10665
HLX	1.083815	3.378866	4.526007	0.001958	0.027794	-1.11289
DSEL	-1.4268	5.001821	-4.51884	0.001976	0.027891	-1.1225
PTPRC	1.420255	7.546544	4.51419	0.001988	0.028013	-1.12876
SHANK3	1.281236	2.134231	4.51251	0.001993	0.028013	-1.13101
CTSS	1.933028	8.781933	4.508246	0.002004	0.028116	-1.13674
NLRC4	1.234748	2.93749	4.50665	0.002008	0.02812	-1.13889
FAM26E	-1.22588	4.265206	-4.50275	0.002018	0.028237	-1.14413
SLCO4A1	2.072865	2.476072	4.50117	0.002023	0.028268	-1.14626
ARRB1	1.140692	5.006204	4.50028	0.002025	0.028273	-1.14746
KCNK13	1.315903	2.121019	4.499085	0.002028	0.02829	-1.14907
DAPP1	1.29576	4.290049	4.490276	0.002052	0.028494	-1.16093
BIRC3	1.143721	4.685273	4.487975	0.002058	0.028494	-1.16403
ITGA6	1.277003	4.990832	4.484506	0.002067	0.028588	-1.16871
CECR1	1.339708	5.96298	4.482057	0.002074	0.028621	-1.17201
CCL19	3.965702	4.331098	4.481079	0.002077	0.028621	-1.17333
PRCP	1.001222	6.662606	4.479719	0.002081	0.028621	-1.17516
HAVCR2	1.678368	5.890071	4.478533	0.002084	0.028621	-1.17676
WHRN	1.891735	2.365196	4.477625	0.002086	0.028627	-1.17798
RSPH10B	-2.34191	1.884063	-4.47593	0.002091	0.028664	-1.18027
AIM1	1.202836	4.052233	4.47487	0.002094	0.028677	-1.1817
FTCDNL1	1.279853	2.332763	4.469441	0.002109	0.028814	-1.18903
PLXNC1	1.189788	5.413326	4.46908	0.00211	0.028814	-1.18952
SAMHD1	1.118635	6.705046	4.466878	0.002116	0.028828	-1.19249
FMO1	1.536896	1.319469	4.465677	0.00212	0.028828	-1.19412
MFSD1	1.136224	7.974234	4.465219	0.002121	0.028828	-1.19473
ITGA4	1.135275	6.55264	4.464506	0.002123	0.028828	-1.1957
APBB1IP	1.209048	4.01346	4.463442	0.002126	0.028837	-1.19714
MMD	1.090834	4.097224	4.461313	0.002132	0.028842	-1.20001
S1PR1	1.630695	4.437134	4.459558	0.002137	0.028842	-1.20239
HEXB	1.074298	7.881436	4.456173	0.002146	0.028842	-1.20696
DAB2	1.325851	7.517758	4.456168	0.002146	0.028842	-1.20697
MAFB	1.582394	5.826657	4.452868	0.002156	0.028914	-1.21143
KRT8	-1.2156	6.053448	-4.44773	0.002171	0.029084	-1.21839
MAPKAPK3	1.133921	5.58362	4.444691	0.002179	0.029163	-1.2225
NR1H3	1.386737	5.445159	4.43821	0.002198	0.029354	-1.23128
RAB42	1.426136	4.211846	4.4345	0.002209	0.029354	-1.23631
THRB	-1.88009	5.272308	-4.43421	0.00221	0.029354	-1.2367
DUSP27	-1.71637	2.514285	-4.43342	0.002212	0.029354	-1.23778
KIAA0226L	1.23981	4.47027	4.433284	0.002213	0.029354	-1.23796

DCN	2.921786	8.669741	4.433149	0.002213	0.029354	-1.23814
COL13A1	-1.51844	4.271122	-4.43303	0.002213	0.029354	-1.23831
DES	-1.96589	4.2682	-4.43043	0.002221	0.029358	-1.24183
ACE	1.529596	3.300525	4.429857	0.002223	0.029358	-1.24261
DOK3	1.502499	4.910833	4.429438	0.002224	0.029358	-1.24318
SIGLEC9	1.418391	4.078759	4.426331	0.002233	0.029431	-1.24739
RAP2B	1.164917	5.167743	4.426165	0.002234	0.029431	-1.24762
SLA	1.502438	5.726246	4.425048	0.002237	0.029434	-1.24914
PILRA	1.83963	5.4237	4.4247	0.002238	0.029434	-1.24961
FNDC5	-1.45636	2.510687	-4.42205	0.002246	0.029457	-1.25321
MYD88	1.3591	4.91863	4.421175	0.002248	0.029464	-1.25439
SUSD5	-2.73597	6.345679	-4.41555	0.002265	0.029539	-1.26204
FRK	-1.33618	3.859386	-4.41372	0.002271	0.029539	-1.26452
PYCARD	1.267966	6.094982	4.413529	0.002271	0.029539	-1.26478
TSPAN15	1.713194	5.038299	4.413319	0.002272	0.029539	-1.26507
ID3	-1.341	8.092713	-4.41326	0.002272	0.029539	-1.26514
SIRPA	1.332936	5.314194	4.412555	0.002274	0.029539	-1.2661
SLC22A17	-1.09023	4.599904	-4.41015	0.002282	0.029539	-1.26937
SPSB1	-1.03753	5.373562	-4.4101	0.002282	0.029539	-1.26945
FAM180A	-1.37376	4.774087	-4.40917	0.002285	0.029539	-1.27071
CD55	-1.35142	6.693056	-4.39162	0.002339	0.030108	-1.2946
KCNAB2	1.722066	5.265761	4.390828	0.002341	0.030108	-1.29567
SH3BGRL3	1.021952	6.586156	4.389069	0.002347	0.030143	-1.29807
GPR137B	1.313509	5.458893	4.378882	0.002379	0.030473	-1.31196
GPNMB	1.240202	9.123354	4.375499	0.002389	0.030556	-1.31658
ANKRD37	-1.30477	5.764113	-4.37461	0.002392	0.030565	-1.3178
AKAP6	-1.33239	6.262531	-4.37331	0.002396	0.030581	-1.31956
ID4	-2.14087	7.135667	-4.36664	0.002418	0.030708	-1.32868
SPI1	1.56363	6.070434	4.366456	0.002419	0.030708	-1.32893
AFAP1L1	1.366685	3.348168	4.363107	0.002429	0.030818	-1.3335
TPRG1	1.403327	4.432781	4.35962	0.002441	0.030844	-1.33827
AQP9	3.530936	3.577055	4.358963	0.002443	0.030844	-1.33917
LYZ	2.017923	8.678493	4.355483	0.002454	0.030915	-1.34393
FCGRT	1.306451	7.899422	4.353105	0.002462	0.030959	-1.34718
AJUBA	-1.22499	3.893222	-4.34953	0.002474	0.031053	-1.35208
C3orf70	-1.4139	1.814738	-4.34681	0.002483	0.031071	-1.35579
MAP2	-1.20622	4.9279	-4.34648	0.002484	0.031071	-1.35625
CTSD	1.710269	10.38223	4.345035	0.002489	0.0311	-1.35823
FGF7	1.712264	6.672264	4.344254	0.002492	0.0311	-1.3593
CERKL	1.0735	3.695043	4.343814	0.002493	0.0311	-1.3599
OSCAR	2.195104	3.966308	4.342095	0.002499	0.031145	-1.36226
LAMA5	-1.19069	5.424688	-4.34097	0.002503	0.031165	-1.3638
ALDH1L1	-1.37144	3.107298	-4.33855	0.002511	0.031239	-1.36712
CD109	1.190268	5.561595	4.333051	0.002529	0.031388	-1.37466
ROR1	-1.75937	3.842886	-4.33085	0.002537	0.031426	-1.37767
PLPP4	1.492268	2.197623	4.326356	0.002552	0.031585	-1.38384
LCP2	1.293776	6.42571	4.324731	0.002558	0.031585	-1.38607
FAM213B	1.256886	3.411319	4.324509	0.002558	0.031585	-1.38638
WWP2	-1.0399	6.456154	-4.3233	0.002563	0.031585	-1.38803
UBASH3B	1.586097	3.766133	4.323236	0.002563	0.031585	-1.38812
NECTIN4	1.796451	1.599475	4.321106	0.00257	0.031648	-1.39105

HSD3B7	1.075824	4.766205	4.318647	0.002579	0.031726	-1.39443
C1QTNF7	-1.69897	4.693464	-4.3167	0.002585	0.031781	-1.39709
SPDYA	-1.00654	2.877683	-4.31593	0.002588	0.031787	-1.39816
HMOX1	2.717043	6.53063	4.312707	0.002599	0.0318	-1.40259
KCNK7	-1.22761	1.425064	-4.30913	0.002612	0.03186	-1.40751
MICU3	-1.07212	4.243212	-4.30708	0.002619	0.031904	-1.41032
NFIX	-1.10298	5.888977	-4.30299	0.002634	0.032015	-1.41595
MAN2B1	1.099149	6.981528	4.300276	0.002643	0.03205	-1.41969
STON2	1.581485	3.500414	4.285459	0.002697	0.032449	-1.4401
CYBB	1.441602	7.218454	4.28013	0.002716	0.032546	-1.44745
PTTG2	-1.00057	1.169946	-4.27601	0.002731	0.032673	-1.45313
KRT16	-2.5336	4.515636	-4.2734	0.002741	0.03271	-1.45673
ACTC1	-3.32622	3.919265	-4.27331	0.002741	0.03271	-1.45686
ZEB1	-1.02121	6.876089	-4.2696	0.002755	0.032847	-1.46198
MEIS1	-1.49102	4.967296	-4.26292	0.00278	0.033008	-1.47122
UNC13D	1.094933	4.701633	4.258339	0.002797	0.033173	-1.47754
DUSP15	-1.13446	1.63493	-4.25801	0.002798	0.033173	-1.478
KALRN	-1.03536	7.596883	-4.2562	0.002805	0.033212	-1.48051
CD53	1.563389	7.462256	4.255822	0.002807	0.033212	-1.48103
ADGRF5	2.722218	3.774838	4.253118	0.002817	0.033224	-1.48477
PLAUR	2.161309	6.790148	4.252106	0.002821	0.033224	-1.48617
ITGAX	1.917623	6.180707	4.251586	0.002823	0.033224	-1.48689
EGLN3	1.104385	3.987647	4.248004	0.002837	0.033288	-1.49184
SMIM10	-1.1608	3.887876	-4.23315	0.002894	0.033749	-1.51243
EXO1	1.070549	1.548478	4.231201	0.002902	0.033806	-1.51513
MYLK	-1.38221	7.941646	-4.22924	0.00291	0.033846	-1.51785
TMEM240	-1.14798	2.319649	-4.22669	0.00292	0.033911	-1.52138
TMEM71	1.411953	3.394656	4.226624	0.00292	0.033911	-1.52148
SHISA4	-1.03062	4.858192	-4.22278	0.002935	0.034033	-1.52681
ROR2	1.283199	2.082532	4.213218	0.002974	0.034421	-1.54009
ATP10A	-1.1396	4.760805	-4.21205	0.002978	0.034421	-1.54172
KLRG1	1.199231	3.294259	4.210847	0.002983	0.03445	-1.54339
BRINP1	-1.66138	2.52452	-4.20922	0.00299	0.03447	-1.54564
CCDC109B	1.306981	5.325617	4.203162	0.003015	0.034583	-1.55407
SHC4	-1.26147	4.706786	-4.2021	0.003019	0.034583	-1.55555
GREB1L	-1.29372	1.742077	-4.20018	0.003027	0.03459	-1.55822
MITF	1.075802	4.780354	4.197583	0.003038	0.034611	-1.56184
ITGB2	1.963587	8.171052	4.19582	0.003045	0.034611	-1.56429
EMCN	2.543853	3.696145	4.195453	0.003047	0.034611	-1.5648
SPINT2	-1.07845	7.55462	-4.19538	0.003047	0.034611	-1.56491
CD86	1.602385	5.485092	4.194481	0.003051	0.034611	-1.56616
RFX8	1.220815	1.456213	4.193108	0.003056	0.034648	-1.56807
RTN4RL1	1.277056	1.890211	4.191724	0.003062	0.034664	-1.56999
STK32A	-1.59152	3.087033	-4.19161	0.003063	0.034664	-1.57016
ATP9A	-1.29485	4.761941	-4.18983	0.00307	0.03472	-1.57264
CYP7B1	1.078587	3.579853	4.188661	0.003075	0.034721	-1.57426
SELPLG	1.379371	4.756164	4.186125	0.003086	0.034809	-1.5778
RYR2	-2.39972	2.576958	-4.18562	0.003088	0.034809	-1.5785
SLC25A19	1.09026	3.938328	4.183722	0.003096	0.034809	-1.58115
TLR2	1.186605	5.9462	4.183602	0.003096	0.034809	-1.58131
NRROS	1.654155	3.657153	4.179616	0.003113	0.034898	-1.58687

SIRPB2	1.624384	4.045708	4.176504	0.003126	0.034925	-1.59121
GP1BB	-1.59427	2.319729	-4.17637	0.003127	0.034925	-1.5914
BMP2K	1.018858	6.664823	4.173189	0.003141	0.035019	-1.59584
HEATR3	1.003774	4.658436	4.172124	0.003145	0.035042	-1.59732
RBM24	-1.06861	1.244409	-4.17088	0.00315	0.035049	-1.59906
KLHL42	-1.28613	5.038112	-4.1697	0.003156	0.035049	-1.60071
PELO	-1.09662	5.966554	-4.16784	0.003164	0.035073	-1.60331
LFNG	1.464161	4.597719	4.16682	0.003168	0.035078	-1.60473
LDOC1	-1.98738	3.890994	-4.16481	0.003177	0.035129	-1.60753
PLAU	1.534729	6.555553	4.164626	0.003177	0.035129	-1.60779
ARL11	1.019284	2.196545	4.160421	0.003196	0.035192	-1.61367
SLC43A3	1.377273	6.142904	4.158637	0.003204	0.035227	-1.61616
C5AR2	1.249561	3.327871	4.154475	0.003222	0.035335	-1.62198
DACT1	-1.38017	5.034348	-4.1542	0.003223	0.035335	-1.62237
CDRT4	-1.18273	3.059898	-4.15313	0.003228	0.035335	-1.62386
FTH1	1.432882	10.69359	4.148478	0.003248	0.035448	-1.63037
C12orf75	-1.55541	7.136019	-4.14369	0.00327	0.035571	-1.63707
CADM1	1.618504	4.968216	4.14222	0.003276	0.035571	-1.63913
LGI2	1.320855	2.977021	4.141954	0.003278	0.035571	-1.6395
CLDN7	1.403367	2.760022	4.141462	0.00328	0.035571	-1.64019
ENAH	-1.07951	6.631235	-4.14083	0.003283	0.035571	-1.64108
FRAS1	-1.47326	1.835386	-4.1408	0.003283	0.035571	-1.64111
CCDC3	-1.0483	6.671248	-4.14055	0.003284	0.035571	-1.64146
CSF1	1.20753	6.146279	4.137671	0.003297	0.035623	-1.6455
PALM2-AKA	-1.05864	4.352745	-4.13244	0.003321	0.035744	-1.65283
ST14	2.030557	4.262037	4.12966	0.003333	0.035796	-1.65673
EFNA1	1.172944	2.87732	4.129343	0.003335	0.035796	-1.65717
TTLL11	-1.04976	3.285175	-4.12904	0.003336	0.035796	-1.6576
C7	2.63873	4.150885	4.128937	0.003337	0.035796	-1.65774
CASQ2	-2.72776	2.477296	-4.12777	0.003342	0.035812	-1.65938
CLEC4A	1.040342	3.889857	4.123668	0.003361	0.035906	-1.66513
PLS3	-1.33351	9.200382	-4.12115	0.003373	0.03595	-1.66866
ITGA7	-1.42676	5.591167	-4.12015	0.003377	0.035973	-1.67006
PLK4	1.049157	2.337632	4.119417	0.003381	0.035982	-1.6711
IFI44	1.250077	5.953751	4.118161	0.003386	0.036017	-1.67286
TNFRSF6B	2.04123	3.461347	4.116426	0.003394	0.036077	-1.67529
CENPN	1.064771	2.809034	4.109174	0.003429	0.03633	-1.68548
ACRBP	1.006912	2.371682	4.102792	0.003459	0.036592	-1.69445
CHI3L1	3.469435	4.285361	4.101035	0.003467	0.036592	-1.69692
BVES	-1.10444	2.449281	-4.10083	0.003468	0.036592	-1.6972
SIGLEC7	1.616749	3.429918	4.098952	0.003477	0.036604	-1.69985
CCL14	2.58224	3.973123	4.098869	0.003477	0.036604	-1.69997
CD163	2.238985	7.325762	4.097564	0.003484	0.036616	-1.7018
SYNC	-1.73997	4.092348	-4.09598	0.003491	0.036669	-1.70404
ITGA11	-1.27424	6.742314	-4.09324	0.003505	0.036781	-1.70789
SLIT3	-1.37393	4.83392	-4.09012	0.00352	0.036831	-1.71228
FAM124B	1.5305	1.521633	4.087207	0.003534	0.036881	-1.71638
HPD	-1.47225	1.992828	-4.08648	0.003537	0.036881	-1.7174
STAC	-1.68026	3.394983	-4.08494	0.003545	0.036906	-1.71958
DOCK8	1.268092	5.481039	4.084236	0.003548	0.036915	-1.72056
GPRC5C	-1.10165	5.336415	-4.08136	0.003562	0.036981	-1.72462

FANCA	1.004161	3.179475	4.080509	0.003567	0.036995	-1.72582
DENND1C	1.228551	2.575242	4.079003	0.003574	0.036995	-1.72794
PTPRE	1.460429	5.542071	4.078524	0.003576	0.036995	-1.72861
BCL6B	1.994458	2.973535	4.07844	0.003577	0.036995	-1.72873
SLC15A3	1.265576	6.381802	4.077828	0.00358	0.037	-1.72959
PARVB	1.350134	4.993814	4.077209	0.003583	0.037005	-1.73047
HEYL	-1.48759	4.497918	-4.0762	0.003588	0.037013	-1.73188
CLDN1	1.48553	4.795969	4.07492	0.003594	0.037013	-1.73369
SNX20	1.036634	3.537186	4.07471	0.003595	0.037013	-1.73399
LIN7A	1.144668	1.489202	4.070099	0.003618	0.037054	-1.74049
TTYH3	1.406671	5.403741	4.069999	0.003619	0.037054	-1.74064
OGN	-1.56388	8.690325	-4.06625	0.003638	0.037113	-1.74593
COL24A1	1.07651	3.295684	4.064184	0.003648	0.037113	-1.74884
CD37	1.266619	5.24058	4.063683	0.00365	0.037113	-1.74955
TLR1	1.367252	6.376912	4.062749	0.003655	0.037113	-1.75087
PCSK6	2.601602	3.722338	4.06212	0.003658	0.037113	-1.75176
CXCL16	1.785829	6.566944	4.057276	0.003683	0.037284	-1.7586
PRSS36	1.108543	2.726924	4.046474	0.003738	0.037711	-1.77386
TRIB1	1.020552	4.059537	4.043073	0.003756	0.037822	-1.77867
IKZF1	1.120407	5.066549	4.03985	0.003773	0.037937	-1.78324
CSPG4	-1.36478	6.255288	-4.03958	0.003774	0.037937	-1.78362
CYTH1	1.094577	5.742395	4.039098	0.003777	0.037937	-1.7843
CSTA	1.370993	3.813797	4.034435	0.003801	0.038103	-1.7909
PPP1R3C	-1.85164	7.019112	-4.03114	0.003819	0.038197	-1.79557
CHMP4C	-2.13604	1.628423	-4.02736	0.003839	0.038371	-1.80093
SRGN	1.587041	8.851999	4.026717	0.003842	0.038378	-1.80183
CCSER1	1.105779	2.273695	4.025588	0.003848	0.038412	-1.80343
LRRC25	1.710489	4.792388	4.024426	0.003854	0.038446	-1.80508
DOCK2	1.376805	6.302115	4.02329	0.00386	0.038454	-1.80669
SLC7A7	1.031146	5.407799	4.018824	0.003884	0.038616	-1.81302
ITGBL1	-1.06011	8.463853	-4.01776	0.00389	0.038616	-1.81454
MTFP1	1.098986	3.045099	4.016672	0.003896	0.038642	-1.81608
SERINC2	1.246998	2.151259	4.015531	0.003902	0.038642	-1.81769
SAMD11	-2.31515	3.286353	-4.00862	0.00394	0.038748	-1.8275
LEFTY2	-2.38128	2.415445	-4.0081	0.003943	0.038748	-1.82824
PPIF	1.161995	4.218598	4.007884	0.003944	0.038748	-1.82855
CDCP1	1.612608	3.557405	4.007562	0.003946	0.038748	-1.829
PTPRJ	1.108618	5.222025	4.003931	0.003966	0.038905	-1.83416
ACKR1	3.119252	2.562233	4.001933	0.003977	0.038966	-1.837
PIK3CG	1.08187	4.042539	3.998852	0.003994	0.039066	-1.84137
CTSC	1.203371	7.452143	3.998025	0.003998	0.039066	-1.84255
TMOD1	-1.29183	3.016834	-3.99593	0.00401	0.039123	-1.84552
RGS19	1.029645	5.01592	3.993983	0.004021	0.039152	-1.8483
SOX6	-1.14315	2.510265	-3.99165	0.004034	0.039226	-1.85162
MAF	1.398548	5.867861	3.99111	0.004037	0.039228	-1.85238
PTPN7	1.163879	3.650725	3.988436	0.004052	0.039294	-1.85619
VAC14	1.047676	4.821849	3.987383	0.004058	0.039325	-1.85768
DSC2	1.243409	3.851662	3.985623	0.004068	0.039359	-1.86019
SLC7A2	1.152553	5.451197	3.983554	0.00408	0.039359	-1.86313
PYGM	-1.41205	2.826835	-3.9823	0.004087	0.03936	-1.86491
UCP2	1.120983	5.741206	3.978612	0.004108	0.039461	-1.87017

MYO5A	1.182794	5.86898	3.975841	0.004124	0.039561	-1.87411
RAB39A	1.273059	2.968798	3.974284	0.004133	0.03962	-1.87633
VEGFC	1.302731	2.855339	3.972688	0.004142	0.039629	-1.8786
ZBTB16	-1.43064	5.705303	-3.96729	0.004173	0.039875	-1.8863
ISYNA1	-1.15174	4.792964	-3.96465	0.004189	0.039916	-1.89006
SYNPO2	-1.83367	6.339985	-3.96463	0.004189	0.039916	-1.89009
CRTAC1	1.444544	6.136274	3.961499	0.004207	0.040011	-1.89456
DNAJB4	-1.09263	6.064117	-3.9585	0.004225	0.040099	-1.89884
SMIM10L2E	-1.01845	1.428129	-3.95581	0.004241	0.040197	-1.90268
PNP	1.377692	5.823539	3.950713	0.004271	0.040412	-1.90995
BCL2A1	2.117201	4.854339	3.946134	0.004299	0.04061	-1.91649
TREM2	1.535826	6.555663	3.939079	0.004341	0.040831	-1.92657
IL12RB2	1.103619	1.639808	3.938947	0.004342	0.040831	-1.92676
IKBKE	1.05904	3.477563	3.935895	0.004361	0.040874	-1.93112
LONRF2	-1.46657	1.750235	-3.93318	0.004377	0.040979	-1.935
HCFC1R1	-1.01057	6.922829	-3.93166	0.004387	0.041034	-1.93718
THSD7B	-1.64934	1.933788	-3.92992	0.004397	0.041081	-1.93967
PGD	1.645986	6.097647	3.928135	0.004408	0.041156	-1.94222
KLHL6	1.265831	4.217052	3.926908	0.004416	0.0412	-1.94398
BHLHB9	-1.07787	2.177425	-3.91888	0.004466	0.041477	-1.95548
CCDC18	1.039365	3.326126	3.917877	0.004472	0.041509	-1.95691
LAIR1	1.457808	6.57675	3.912342	0.004507	0.041654	-1.96484
BAIAP2	1.288213	3.438451	3.91215	0.004508	0.041654	-1.96512
MSR1	1.815434	8.614017	3.909931	0.004522	0.04173	-1.9683
FLT1	2.019654	4.052404	3.906703	0.004543	0.04183	-1.97293
FCGR1A	1.321791	7.919496	3.905482	0.004551	0.04183	-1.97468
C11orf96	-1.87962	7.001697	-3.90297	0.004567	0.041915	-1.97828
GIMAP7	1.301295	5.017763	3.902372	0.004571	0.041915	-1.97914
GPX1	1.329742	8.965301	3.902199	0.004572	0.041915	-1.97939
BEX5	-1.17045	2.789613	-3.89875	0.004594	0.042027	-1.98434
HMGB3	1.270691	3.023556	3.897832	0.0046	0.042027	-1.98565
CYGB	1.437491	2.722446	3.897129	0.004605	0.042027	-1.98666
SLC2A12	-1.33705	2.260117	-3.89668	0.004607	0.042027	-1.98731
IGFBP4	1.60125	5.945023	3.895756	0.004613	0.042027	-1.98863
TNFRSF21	1.73256	4.167612	3.892738	0.004633	0.042169	-1.99297
ZNF366	1.048472	1.392109	3.89204	0.004638	0.042169	-1.99397
C15orf48	2.462107	3.952815	3.889146	0.004657	0.042195	-1.99813
RNF180	-1.06211	4.789678	-3.88888	0.004658	0.042195	-1.9985
MPP7	-1.67593	4.199178	-3.88792	0.004665	0.042223	-1.99988
EMP1	1.117024	6.899627	3.886457	0.004674	0.042284	-2.00199
CD48	1.459662	5.160074	3.881368	0.004708	0.042437	-2.0093
ANLN	1.030318	2.74842	3.881195	0.004709	0.042437	-2.00955
SIGLEC15	1.404265	2.907831	3.875179	0.004749	0.042593	-2.0182
CLIP3	-1.05616	3.203827	-3.87499	0.00475	0.042593	-2.01847
PLN	-2.22699	7.764711	-3.87458	0.004753	0.042593	-2.01906
PRRT1	-1.1656	2.172464	-3.86988	0.004785	0.042795	-2.02582
CYBA	1.306408	9.010052	3.86896	0.004791	0.04281	-2.02715
TMEM132B	-1.55888	1.409926	-3.86875	0.004792	0.04281	-2.02745
FYB	1.190016	5.685877	3.865849	0.004812	0.042878	-2.03163
NUP210	1.119161	3.181893	3.858417	0.004863	0.043222	-2.04233
TUBB3	1.484577	3.856471	3.85465	0.004889	0.043394	-2.04776

PCDH20	-2.19071	1.740336	-3.8533	0.004898	0.043414	-2.04971
HIST1H2AG	1.012899	4.80804	3.850251	0.004919	0.043418	-2.0541
IL17RE	-1.04199	3.429888	-3.84969	0.004923	0.043418	-2.05491
FILIP1L	-1.87097	6.634371	-3.84865	0.00493	0.043418	-2.05641
GRHL2	-1.19785	2.219458	-3.84792	0.004935	0.043418	-2.05746
CARNS1	-1.31886	2.136526	-3.8474	0.004939	0.043418	-2.05821
NDP	1.012309	1.48609	3.841142	0.004983	0.043639	-2.06724
MYOZ1	-1.69177	3.280205	-3.83489	0.005027	0.043892	-2.07626
LRRC4B	-1.68239	2.102379	-3.8307	0.005057	0.044099	-2.08232
ASB2	-1.42342	3.45324	-3.82874	0.005071	0.044168	-2.08515
HOMER1	-1.03381	3.176184	-3.82803	0.005076	0.044185	-2.08618
DNASE1L3	2.352513	3.136559	3.824821	0.005099	0.044335	-2.09081
FAM160A1	-1.01334	2.112781	-3.82478	0.0051	0.044335	-2.09087
TSPAN2	-2.2331	4.958994	-3.82359	0.005108	0.044367	-2.0926
TBL1X	-1.21138	4.299612	-3.82282	0.005114	0.044367	-2.0937
LYN	1.257075	5.506738	3.822293	0.005118	0.044367	-2.09446
SP140	1.194626	3.863644	3.819222	0.00514	0.044521	-2.0989
CD14	1.710992	8.844971	3.81882	0.005143	0.044521	-2.09949
GAS7	1.441402	5.005409	3.817958	0.005149	0.044549	-2.10073
IFI44L	1.183293	5.613984	3.814677	0.005173	0.044648	-2.10548
TMSB15B	-1.37535	3.283431	-3.809	0.005215	0.044833	-2.11369
RGL1	1.081496	4.383797	3.808273	0.00522	0.044839	-2.11475
SLC46A2	1.366645	1.87539	3.80774	0.005224	0.044846	-2.11552
PDE8B	-1.43463	2.579765	-3.8068	0.005231	0.044875	-2.11688
TCEAL2	-1.76909	3.610385	-3.80261	0.005262	0.045	-2.12295
TBXAS1	1.303826	5.887007	3.801866	0.005268	0.045005	-2.12402
PSD4	1.336961	4.330812	3.798838	0.005291	0.045118	-2.12841
TMEM246	-1.0337	3.045682	-3.79832	0.005295	0.045124	-2.12916
SNAP25	-1.48863	1.92565	-3.79729	0.005302	0.045163	-2.13065
HCK	1.34116	5.871509	3.787042	0.00538	0.045602	-2.14551
CCDC81	-1.04992	2.254775	-3.78279	0.005413	0.045729	-2.15168
ARPP21	-1.83913	1.619302	-3.78268	0.005414	0.045729	-2.15184
SLC36A1	1.085706	4.352743	3.7815	0.005423	0.045752	-2.15355
LDLRAD2	-1.58515	3.116803	-3.77819	0.005448	0.0459	-2.15835
ADGRG1	1.225111	2.412503	3.775754	0.005467	0.046001	-2.16189
TLN2	-1.00268	4.466648	-3.77205	0.005496	0.046154	-2.16727
MYCT1	1.412412	2.31184	3.76577	0.005546	0.046486	-2.17639
BCAM	-1.01843	5.444323	-3.76288	0.005568	0.046625	-2.18059
MEOX1	1.427622	1.446494	3.76252	0.005571	0.046625	-2.18111
HCST	1.470637	5.525431	3.762449	0.005572	0.046625	-2.18121
IFI30	1.953535	8.444742	3.759065	0.005599	0.046733	-2.18613
F10	-1.26475	3.288579	-3.75863	0.005602	0.046733	-2.18676
LST1	1.243833	6.253594	3.757468	0.005612	0.046752	-2.18846
LGI1	-1.89243	1.588057	-3.75726	0.005613	0.046752	-2.18876
HYAL2	1.183541	5.164667	3.753854	0.005641	0.046835	-2.19371
NCF2	1.775137	6.033701	3.753766	0.005641	0.046835	-2.19384
JAG1	-1.04322	7.53607	-3.7518	0.005657	0.046898	-2.19669
MME	1.305252	3.132452	3.748087	0.005687	0.047121	-2.2021
NEGR1	-2.02022	4.07545	-3.74245	0.005733	0.047282	-2.21031
ADRA2C	-2.66927	3.104071	-3.74095	0.005745	0.047356	-2.21249
LIMCH1	-1.21012	6.708586	-3.73952	0.005757	0.047396	-2.21457

KANK3	1.453092	2.356099	3.736061	0.005786	0.047501	-2.21962
ART4	-1.33306	4.949825	-3.73599	0.005786	0.047501	-2.21972
CNTN1	-2.77408	3.196548	-3.73241	0.005816	0.04769	-2.22494
TPM3	1.076759	8.605772	3.729291	0.005842	0.047709	-2.22948
KCNJ3	-1.73065	1.778085	-3.72906	0.005844	0.047709	-2.22982
LILRB5	1.818687	3.472774	3.729004	0.005844	0.047709	-2.2299
FPR3	1.779061	6.350707	3.728361	0.00585	0.04772	-2.23084
SLC2A5	2.419012	4.057533	3.727068	0.005861	0.047756	-2.23272
DOK2	1.330857	4.791782	3.724891	0.005879	0.047816	-2.2359
REEP1	-1.83873	2.349346	-3.72425	0.005884	0.047816	-2.23684
VAMP8	1.655188	6.920707	3.71717	0.005944	0.048096	-2.24716
ECM2	-1.37242	7.295028	-3.71069	0.006	0.04839	-2.25663
AIF1	1.166368	7.59819	3.698792	0.006103	0.048946	-2.27401
CTSZ	1.006392	9.646242	3.695463	0.006132	0.049069	-2.27887
CASP1	1.138876	6.300168	3.694081	0.006144	0.049069	-2.2809
RAMP3	1.866568	3.667066	3.693507	0.006149	0.049069	-2.28173
ERBB4	-1.03405	5.234921	-3.69183	0.006164	0.049069	-2.28419
KRBOX1	-1.15425	2.446578	-3.69155	0.006167	0.049069	-2.28459
S1PR4	1.151888	2.645276	3.690948	0.006172	0.049069	-2.28548
CTSG	2.539622	2.970845	3.690858	0.006173	0.049069	-2.28561
EXOC3L2	1.313734	1.566201	3.687717	0.006201	0.04926	-2.2902
ANKS1B	-1.35914	4.256072	-3.68439	0.00623	0.049426	-2.29508
FLVCR2	1.2049	3.853243	3.682087	0.006251	0.049522	-2.29844
GAS2L3	1.176416	4.740432	3.678589	0.006282	0.049662	-2.30357
PLP1	-1.57559	1.796566	-3.6769	0.006298	0.04974	-2.30604
CCDC146	-1.52439	4.286186	-3.67633	0.006303	0.04974	-2.30687
GPSM3	1.202367	5.097371	3.673033	0.006333	0.049922	-2.3117

Abbreviations: FC: fold change; AveExpr: average expression; adj.P.Val: adjust P value
